# Lack of support for Deuterostomia prompts reinterpretation of the first Bilateria

**DOI:** 10.1126/sciadv.abe2741

**Published:** 2021-03-19

**Authors:** Paschalia Kapli, Paschalis Natsidis, Daniel J. Leite, Maximilian Fursman, Nadia Jeffrie, Imran A. Rahman, Hervé Philippe, Richard R. Copley, Maximilian J. Telford

**Affiliations:** 1 Centre for Life’s Origins and Evolution, Department of Genetics, Evolution, and Environment, University College London, Gower Street, London WC1E 6BT, UK.; 2 Oxford University Museum of Natural History, Parks Road, Oxford OX1 3PW, UK.; 3 Station d’Ecologie Théorique et Expérimentale, UMR CNRS 5321, Université Paul Sabatier, 09200 Moulis, France.; 4 Laboratoire de Biologie du Développement de Villefranche-sur-mer (LBDV), Sorbonne Université, CNRS, 06230 Villefranche-sur-mer, France.

## Abstract

The bilaterally symmetric animals (Bilateria) are considered to comprise two monophyletic groups, Protostomia (Ecdysozoa and the Lophotrochozoa) and Deuterostomia (Chordata and the Xenambulacraria). Recent molecular phylogenetic studies have not consistently supported deuterostome monophyly. Here, we compare support for Protostomia and Deuterostomia using multiple, independent phylogenomic datasets. As expected, Protostomia is always strongly supported, especially by longer and higher-quality genes. Support for Deuterostomia, however, is always equivocal and barely higher than support for paraphyletic alternatives. Conditions that cause tree reconstruction errors—inadequate models, short internal branches, faster evolving genes, and unequal branch lengths—coincide with support for monophyletic deuterostomes. Simulation experiments show that support for Deuterostomia could be explained by systematic error. The branch between bilaterian and deuterostome common ancestors is, at best, very short, supporting the idea that the bilaterian ancestor may have been deuterostome-like. Our findings have important implications for the understanding of early animal evolution.

## Teaser

The deuterostome animal groups (Chordata and Xenambulacraria) may not be each other’s closest relatives.

## INTRODUCTION

The bilaterally symmetric animals (Bilateria) are widely held to be composed of two major monophyletic groups, Protostomia and Deuterostomia. Protostomia contains the two branches of Ecdysozoa and Lophotrochozoa; Deuterostomia contains the Chordata, including vertebrates and Xenambulacraria (Hemichordata, Echinodermata, and, more controversially, Xenacoelomorpha). The names Protostomia and Deuterostomia refer to a supposed distinct origin of the mouth in the two clades, but they have been differentiated by other embryological characters including embryonic cleavage patterns and distinct ways of forming their mesoderm and coelomic cavities. While Protostomia is highly supported by molecular data, including numerous unique genomic characters, the deuterostomes have fewer convincing molecular synapomorphies, and some recent studies of metazoan phylogeny do not support deuterostome monophyly. The inconsistent support seen for Deuterostomia in recent studies might be evidence that a rapid and ancient radiation in the Precambrian produced the two deuterostome subclades. Cases of rapid radiations imply a limited time between speciations (short internal branches) that can lead to the accumulation of minimal phylogenetic signal, and this signal can be easily blurred by the numerous substitutions occurring later (long terminal branches). Such problems are exacerbated when heterogeneities in the evolutionary process cause model violations producing systematic errors (*1*). Here, we consider recent indications that the limited support for the major animal superphylum of Deuterostomia may result from these problematic circumstances. We ask whether support from molecular phylogenetic studies for the deuterostome clade, widely accepted for over 100 years, may even be the result of an artifact of tree reconstruction.

## RESULTS

### The deuterostome branch is short and has low support compared to the protostome branch

**Fig. 1:**
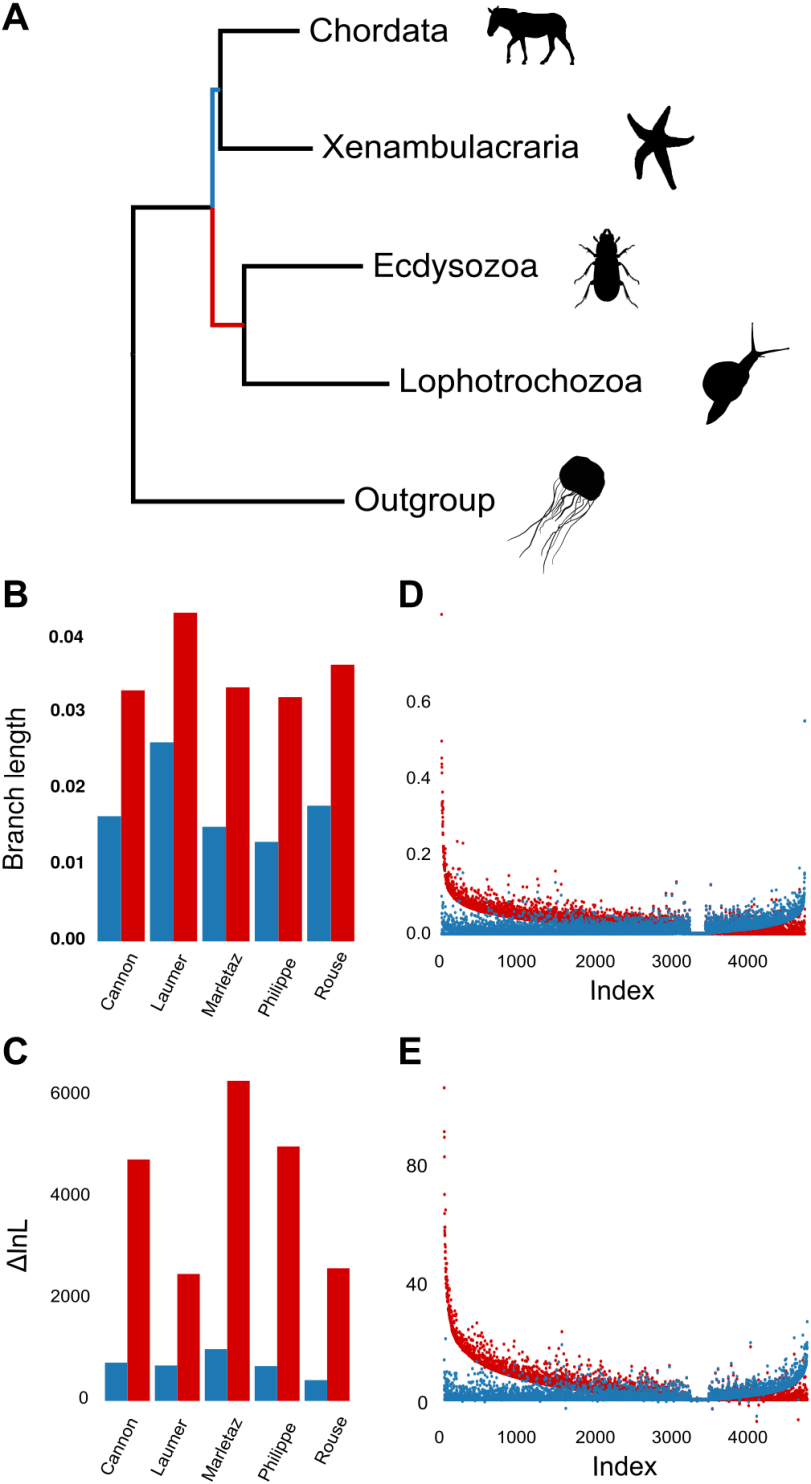
The deuterostome branch is short and weakly supported compared with the protostome branch. (**A**) Canonical tree showing relationships between major metazoan clades. The Bilateria consists of two branches: Protostomia (red, Lophotrochozoa and Ecdysozoa) and Deuterostomia (blue, Chordata and Xenambulacraria). (**B**) Comparison of branch lengths from the bilaterian common ancestor to the deuterostome common ancestor (blue) and to the protostome common ancestor (red). In five datasets, the protostome branch is twice as long as the deuterostome branch. (**C**) Comparison of decrease in lnLikelihood of a tree in which the protostome or deuterostome branch has been collapsed (∆lnL). In five datasets, collapsing the protostome branch reduces lnL considerably more than collapsing the deuterostome branch. (**D**) Comparison of estimated protostome and deuterostome branch lengths for individual genes. Genes from all five datasets are ordered by the difference in length between protostome and deuterostome branches. In 3376 out of 4826 genes (69.9%), the protostome branch is longer. (**E**) Comparison of protostome and deuterostome ∆lnL for individual genes. Genes from all five datasets are ordered by the difference in ∆lnL between protostomes and deuterostomes. In 3312 out of 4826 genes (68.6%), the protostome ∆lnL is larger. All analyses used the LG + F + G model.

The different topologies relating Chordata, Xenambulacraria, and Protostomia seen in recently published animal phylogenies suggest that the support for monophyletic deuterostomes (Fig. 1A) may be weak. This contrasts with consistent, strong support for the monophyly of the protostomes. To study the support for the deuterostome clade in the context of the well-supported protostome clade, we use five recent, independently generated, phylogenomic datasets covering the diversity of animal phyla [i.e., (*2*–*6*)]. We investigate the support for different topologies relating the Chordata, Xenambulacraria, Ecdysozoa, and Lophotrochozoa. We first asked whether the branches leading to protostomes and deuterostomes are of similar length, under the assumption that both are monophyletic. We note that, while the classic phylogeny suggests that protostomes and deuterostomes originated at the same point (Urbilateria), there is no expectation that the branches leading to the common ancestors of Protostomia and Deuterostomia should be of the same length. The point of the comparison is to see whether there is a strong contrast in branch length between the well-supported Protostomia and the less well-supported Deuterostomia.

Using a fixed topology taken from the original publications for each dataset (modified when necessary to enforce monophyly of deuterostomes), we optimized branch lengths using the site-homogeneous LG + F + G model. While the overall amount of change in branches leading from the bilaterian common ancestor to both protostomes and deuterostomes differs between datasets, in all cases, the branch leading to the protostomes is approximately twice the length of the branch leading to the deuterostomes (Fig. 1B.). Under more realistic site heterogeneous models, the protostome branch is almost six times longer. This difference is reinforced when we look at the change in log likelihood (∆lnL) between a fully resolved tree compared with trees in which either the protostome or deuterostome branch is collapsed into a polytomy. The ∆lnL observed on collapsing the branch leading to protostomes is considerably greater for all five datasets than the ∆lnL when collapsing the deuterostome branch (Fig. 1C).

We repeated these measures of branch lengths and ∆lnL using all individual genes from all five datasets (Fig. 1, D and E). For the great majority of genes across all datasets, as for the concatenated datasets, there is a longer branch leading to the protostomes than to the deuterostomes (protostomes longer in 70% of genes) and consistently stronger support (larger ∆lnL) for protostomes than for deuterostomes (∆lnL protostomes larger for 68.6% of genes). Most individual genes support monophyletic deuterostomes much less strongly than they support protostomes.

### A minority of genes support deuterostome monophyly

**Fig. 2:**
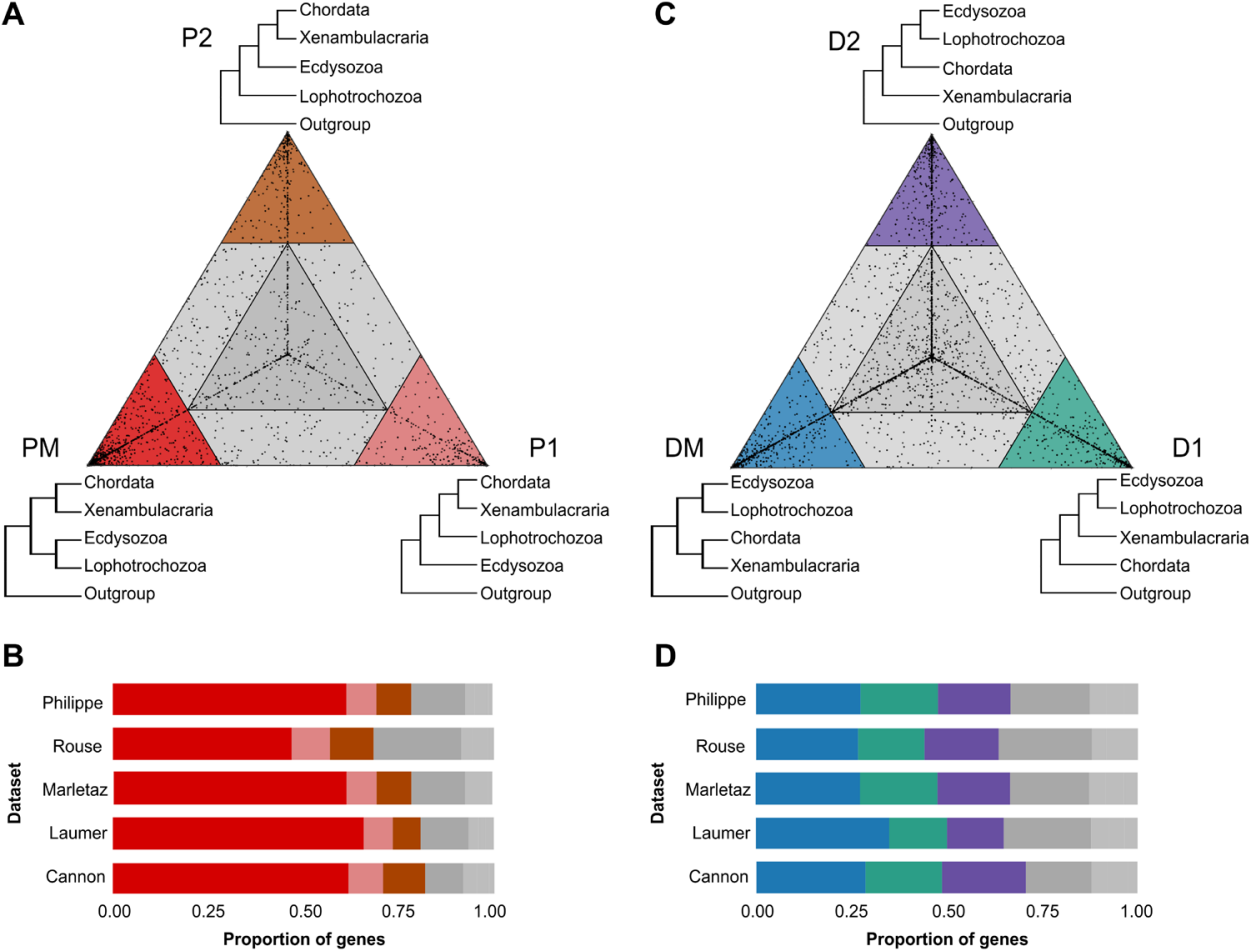
Most genes prefer monophyletic Protostomia over alternatives; few genes prefer monophyletic Deuterostomia. Triangular plots showing relative support for the three alternative topologies shown at the corners of the triangles. Genes in colored corners show a high preference for the corresponding topology. The numbers of genes found in the different colored sectors of the large triangle are shown below. (**A**) Triangle plot comparing support for monophyletic Protostomia (PM) versus two alternative topologies with paraphyletic Protostomia (P1 and P2). (**B**) Bar plot showing that across five datasets, most genes strongly prefer the monophyletic Protostomia topology. (**C**) Triangle plot comparing support for monophyletic Deuterostomia (DM) versus two alternative topologies with paraphyletic Deuterostomia (D1 and D2). (**D**) Bar plot showing that across five datasets, a minority of genes strongly prefer the monophyletic Deuterostomia topology over paraphyletic topologies or the gray areas.

The measures presented above are based on trees in which deuterostomes were constrained to be monophyletic, meaning that we could not show any topological conflict between genes. To measure conflict between genes, we considered each gene individually and compared the lnLikelihood of a tree supporting protostome monophyly (“PM”) or deuterostome monophyly (“DM”) with the lnLikelihoods of two alternative topologies for each of these two clades: protostome paraphyly “P1”: Lophotrochozoa sister to Deuterostomia; protostome paraphyly “P2”: Ecdysozoa sister to Deuterostomia; deuterostome paraphyly “D1”: Xenambulacraria sister to Protostomia; and deuterostome paraphyly “D2”: Chordata sister to Protostomia (see Fig. 2).

We visualized the relative lnLikelihoods for the three topologies for every gene on a triangular plot (Fig. 2). For the protostome trees, most genes have a strong preference for a single topology (77.31% on average across datasets with the LG + F + G model); the majority of genes support the monophyly of protostomes (58.94% versus 9.71% and 8.66% for PM, P1, and P2, respectively; table S1).

For the deuterostomes, the results are more equivocal; fewer genes have strong preference for a single topology (66.99% on average), and there is only a small excess in the number of genes strongly supporting monophyletic deuterostomes over the other two topologies (28.65% versus 19.17% and 19.17% for DM, D1, and D2, respectively; table S1).

To address potential questions regarding the monophyly of the Xenambulacraria, these individual gene analyses were repeated using datasets from which the Xenacoelomorph *Xenoturbella* had been removed. The removal of this taxon resulted in subtle changes to the results but did not change any conclusions (see fig. S1).

For all five datasets, the gene alignments strongly supporting the monophyletic protostomes topology were longer and had higher monophyly scores (a measure of their ability to support known clades) compared with the gene alignments that supported either of the two alternative topologies (fig. S2). For the deuterostomes, there was minimal evidence of a significant difference of alignment length between genes supporting DM versus either paraphyletic alternative (fig. S2), and there were no significant differences in monophyly scores between genes supporting DM versus either paraphyletic alternatives (table S3). Monophyletic Protostomia, but not monophyletic Deuterostomia, is strongly preferred across five independent datasets by a clear majority of individual genes that are, on average, longer with a stronger phylogenetic signal.

### DM is correlated with conditions that may lead to systematic error

Whatever the true topology relating Xenambulacraria, Chordata, and Protostomia, we have shown that the branch separating them is short and might therefore be especially sensitive to systematic errors. Systematic errors can result from heterogeneities in the process of substitution that are not accommodated by the models used and can be exacerbated in faster evolving datasets. In the context of unequal rates of evolution among taxa, this may lead to a long-branch attraction (LBA) artifact (*7*).

**Fig. 3:**
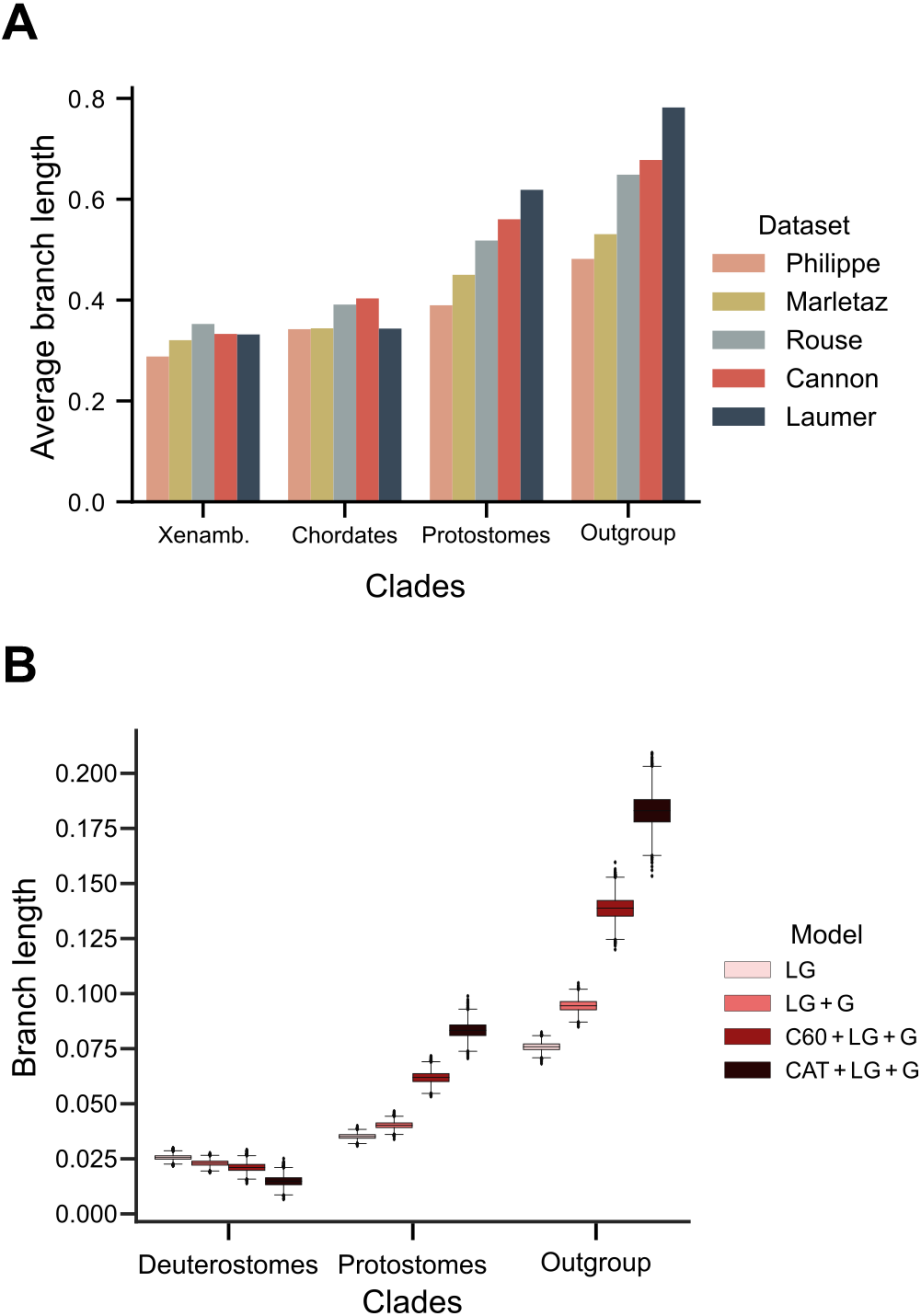
Evidence from empirical data that conditions promoting systematic error correlate with support for monophyletic Deuterostomia. (**A**) Bar chart showing the average branch lengths within Chordata, Xenambulacraria, Protostomia, and from the outgroup taxa to bilaterian root. Datasets with the greatest branch length heterogeneity (Rouse, Cannon, and Laumer) support deuterostome monophyly. (**B**) Box plot showing the lengths of branches leading to the Deuterostomia, Protostomia, and from the outgroups to Bilateria estimated using different models of evolution with increasing fit to data. With improving model fit, the estimated length of the deuterostome branch decreases, and estimated protostome and outgroup branch lengths increase—conditions that could reduce LBA between protostome and outgroups.

For each dataset, we measured the average branch lengths within the protostome, chordate, and xenambulacrarian clades (average distance from the bilaterian common ancestor to each taxon within each clade) and the average distance from each outgroup taxon to the bilaterian common ancestor. The branches leading to the outgroups and to the protostomes are always longer than the branch leading to the chordate and xenambulacrarian clades (Fig. 3A). Under LBA, the long-branched protostomes and outgroup might be expected to be mutually attracted, resulting in clustering of short-branched Chordata and Xenambulacraria.

The three datasets that supported monophyletic deuterostomes (*2*, *3*, *6*) had taxon samples that resulted in the three largest rate inequalities with the longest average protostome and outgroup branch lengths relative to chordate and xenambulacrarian branches (Fig. 3A). The three datasets that support monophyletic Deuterostomia are also the fastest evolving (expected substitutions per site measured across a tree containing the eight taxa that all five datasets have in common: Philippe, 1.971; Marletaz, 2.2082; Cannon, 2.537; Laumer, 2.6578; and Rouse, 2.8741).

Last, while two datasets support paraphyletic deuterostomes under complex site heterogeneous models (*4*, *5*), which account for across-site amino acid preference variability (*8*), all five datasets support monophyletic deuterostomes when using simpler and less well-fitting site homogeneous models. Unequal rates, faster-evolving loci, and inadequate site homogeneous models are all known to promote LBA artifacts, especially in the context of short internal nodes (*1*, *7*, *9*). All these problem-causing conditions correlate with support for Deuterostomia.

### Less well-fitting models produce conditions that may bias toward monophyletic deuterostomes

We further examined the possible effect of model misspecification by comparing branch lengths estimated using different models. We used the Laumer *et al*. dataset (*3*), which has the clearest branch length heterogeneity (Fig. 3A). We estimated the lengths of the branches leading to the deuterostomes, the protostomes, and the outgroup. We see that as we use models that fit the data increasingly well, the estimated length of the branch leading to the deuterostomes decreases, and the lengths of the branches leading to the protostomes and outgroup increase (Fig. 3B). Using inadequate models will underestimate the likelihood of convergence between Protostomia and outgroup, possibly resulting in an LBA between protostomes and outgroups.

### Simulations show that the monophyletic deuterostome topology is favored under conditions producing systematic errors

**Fig. 4:**
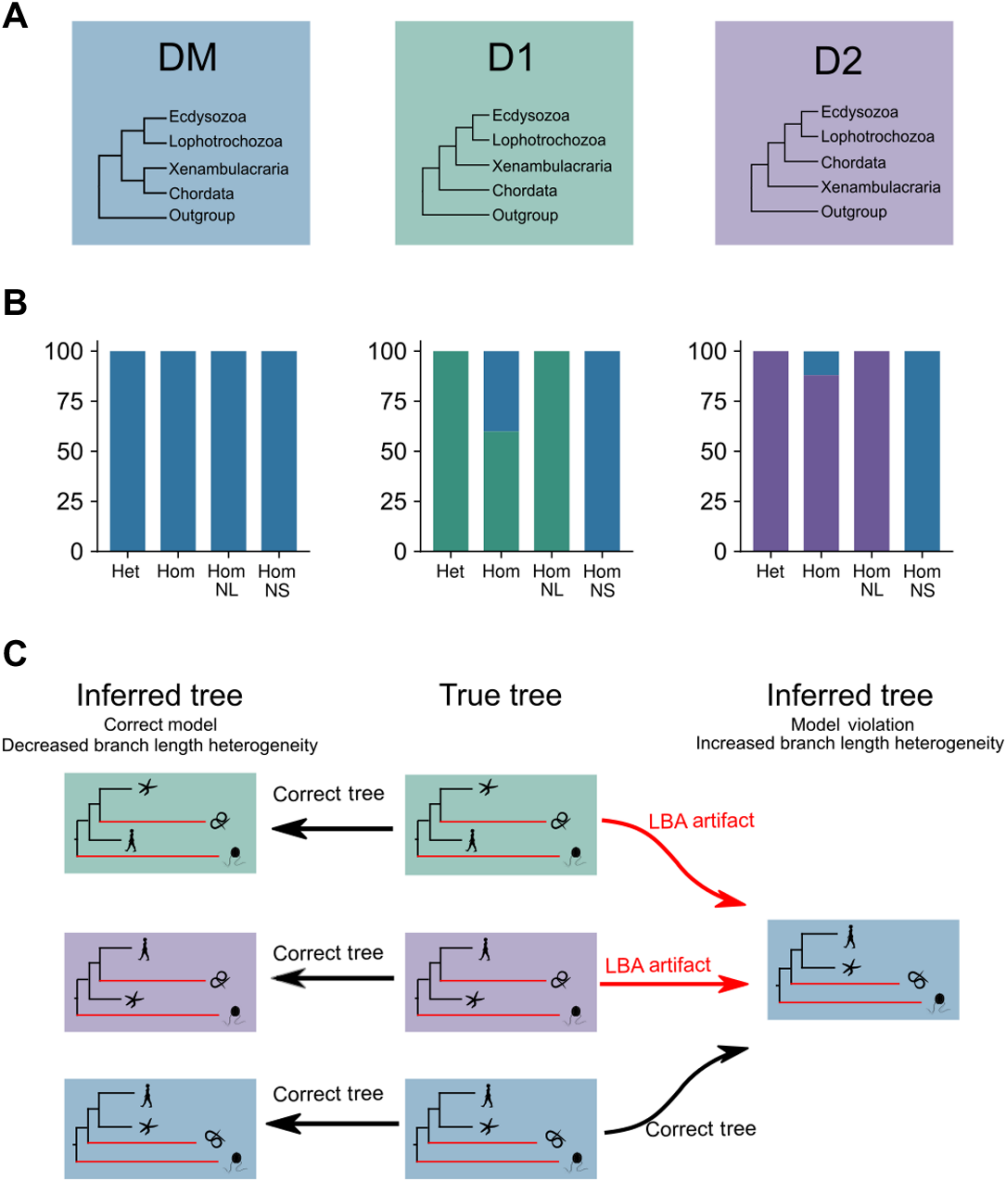
Simulations show that model violations and unequal rates result in artifactual support for monophyletic Deuterostomia. (**A**) One hundred datasets were simulated using parameters estimated under a site heterogeneous model (CAT + LG + G) for each of the three topologies shown (colored boxes). (**B**) For each simulated dataset, a maximum likelihood tree was reconstructed under four conditions: i) site heterogeneous C60 + LG + F + G model (Het); ii) model violating site homogeneous LG + F + G model (Hom); iii) homogeneous model with long branches of protostomes and outgroup taxa removed (HomNL); iv) homogeneous model with short-branch protostomes and outgroup taxa removed (HomNS). For each condition, the number of times the three possible topologies were reconstructed is shown in the bar charts; color indicates the topology supported. Data simulated under DM always yield the correct topology. Under the site heterogeneous model, D1 and D2 data always yield a correct topology. Under the model violating site homogeneous model, D1 and D2 data yield an incorrect topology in 40% and 12% of replicates, respectively. The incorrect tree is always DM. Under the site homogeneous model with long-branch protostomes and outgroups removed (reducing LBA), D1 and D2 data always yield a correct topology. Under the site homogeneous model with short-branch protostomes and outgroups removed (enhancing LBA), D1 and D2 data always yield an incorrect topology. The incorrect tree is always DM. (**C**) Interpretation of simulation results as an LBA artifact. The tree topologies under which data were simulated (true tree) are shown in the middle. The long branches leading to the protostomes and outgroups are indicated in red. Under conditions that minimize systematic error (left, site heterogeneous model or reduction in branch length heterogeneity by removing long branches), the correct tree (DM, D1, or D2) is always reconstructed. Under conditions that enhance systematic error (right, model violating site homogeneous models or increase in branch length heterogeneity by removing short branches), the DM topology is reconstructed using data simulated under all three topologies.

We next followed a data simulation approach to ask whether any of the three topologies relating the Protostomia, Chordata, and Xenambulacraria (Fig. 4A) could gain artifactual support as a result of systematic error. Using 50,000 positions and 36 taxa from the Laumer dataset, we estimated parameters using the three alternative topologies relating the Chordata, Xenambulacraria, and Protostomia (DM, D1, and D2; Fig. 4A) under the best-fitting CAT + LG + G model implemented in phylobayes (*10*) . For each topology, we simulated 100 datasets using these estimated parameters.

For data simulated according to the DM topology, we are able to reconstruct monophyletic deuterostomes (DM) correctly in 100% of replicates under both correct (site heterogeneous) and model violating (site homogeneous models) (Fig. 4B). In contrast, for data simulated under the two topologies with paraphyletic deuterostomes, recovering the true relationships was more challenging. As predicted by the consistency of probabilistic models, under the correct, site heterogeneous model, the correct paraphyletic topology was always recovered. Under the model-violating site homogeneous model (expected to exacerbate LBA artifacts), the true tree was recovered in 60 and 88% of the datasets simulated under the D1 and D2 hypotheses, respectively (Fig. 4B). In both cases, the remaining 40% (D1) and 12% (D2) of the datasets incorrectly recovered monophyletic deuterostomes (the DM topology).

These analyses were repeated using a dataset from which the Xenacoelomorph *Xenoturbella* had been removed. The removal of this taxon resulted in subtle changes to the results that do not affect the conclusions drawn (see table S4).

### Branch length heterogeneity is correlated with support for monophyletic deuterostomes

To explore the hypothesis of unequal rates of evolution lending exaggerated support to the monophyletic Deuterostomia topology, we reinferred trees using the same simulated data after first removing the 13 longest branched taxa of protostome and outgroup clades (*11*, *12*). We reinferred the trees using the site homogeneous LG + F + G model, which had resulted in some incorrect topologies with the full dataset. Regardless of the topology under which the data were simulated, removing these long branches resulted in recovering the correct topology in 100% of cases (Fig. 4B).

We contrast this with an equivalent experiment designed to exaggerate artifacts caused by rate heterogeneity. We removed the 13 shortest protostome and outgroup taxa from the simulated data and again reinferred the tree topologies under the site homogeneous LG + F + G model. Regardless of the topology under which the data were simulated, we recovered monophyly of the deuterostomes (DM topology) in 100% of replicates (Fig. 4B). Our results show that if deuterostomes are paraphyletic (either paraphyletic topology), then conditions we have shown to affect real datasets could easily result in artifactual support for monophyletic deuterostomes (Fig. 4C and table S4).

### Reappraising deuterostome molecular synapomorphies

Studies of different classes of molecular characters also suggest stronger support for Prostostomia than for Deuterostomia. Protostomes have 12 unique microRNA families (*13*) compared with 1 in deuterostomes, and protostomes share 58 “near intron pairs” compared with only 7 in deuterostomes. Protostomes also share a highly distinct, conserved variant of the mitochondrial NAD5 protein (*14*) and a hidden break in their 28*S* ribosomal RNA (*15*).

In their comparison of hemichordate and chordate genomes, Simakov *et al*. (*16*) conducted a systematic search for genes unique to deuterostomes. Using the greater number and diversity of sequenced genomes now available, we have identified likely orthologs of many of these genes in nonbilaterian metazoans and/or in protostomes. Overall, we have found evidence for 20 out of 31 of these “deuterostome novelties” in protostomes and/or nonbilaterian metazoans, suggesting that they are bilaterian plesiomorphies (Supplementary Text). Equivalent searches for characters unique to either Chordata plus Protostomia or Xenambulacraria plus Protostomia have not been conducted, and it is, therefore, not clear how to interpret the 11 remaining Chordata plus Xenambulacraria–specific characters. If deuterostomes are not monophyletic, then these 11 genes must have been lost in protostomes.

### The pharyngeal gene cluster may be a bilaterian, not a deuterostomian character

**Fig. 5:**
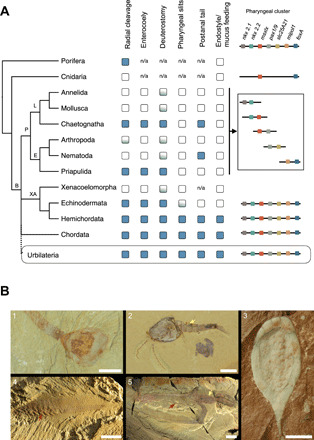
Character evolution implied by a short or nonexistent deuterostome branch. (**A**) Distribution of characters on a phylogenetic tree with an unresolved polytomy at the base of the Bilateria involving Protostomia (P), Xenambulacraria (XA), and Chordata. (L, Lophotrochozoa; E, Ecdysozoa; B, Bilateria). Presence or absence of the characters discussed in the Discussion are indicated (blue box, present; white box, absent; blue/white box, variable; n/a, not applicable). The pharyngeal cluster contains the seven genes indicated by colored boxes. The cluster has not been found intact in any single protostome, but most possible pairs/triplets of adjacent genes are found linked in one or more protostomes or nonbilaterian metazoans (box represents various protostomes; see text for details), implying that the common ancestor of protostomes had an intact cluster. Urbilateria is the common ancestor of all three clades (dotted line), and its characteristics can be inferred as those present in Chordata and Xenambulacraria (and, for some characters, in Protostomia). (**B**) Cambrian bilaterians. 1, The lophotrochozoan *Lingulella chengjiangensis* (Cambrian Series 2, Yunnan Province, China). 2, The ecdysozoan *Chuandianella ovata* (Cambrian Series 2, Yunnan Province, China). 3, The xenambulacrarian *Protocinctus mansillaensis* (Cambrian Series 3, Spain). 4, The chordate *Myllokunmingia fengjiaoa* (Cambrian Series 2, Yunnan Province, China). 5, The problematic bilaterian *Vetulicola cuneata* (Cambrian Series 2, Yunnan Province, China). Pharyngeal slits (red arrows) are present in *Vetulicola* and *Myllokunmingia*, while a segmented bipartite body (yellow arrows) is a feature of *Vetulicola* and *Chuandianella*. If Urbilateria had pharyngeal slits, then *Vetulicola* could represent a stem protostome. Images 1, 2, 4, and 5 are courtesy of Yunnan Key Laboratory for Palaeobiology and MEC International Joint Laboratory for Palaeobiology and Palaeoenvironment, Yunnan University, Kunming, China. Image 3 is courtesy of S. Zamora. Scale bars, 5 mm (1 to 3) and 10 mm (4 and 5).

Simakov *et al*. (*16*) also describe a deuterostomian “pharyngeal gene cluster.” This is a microsyntenic block of seven genes (*nkx2.1*, *nkx2.2*, *msxlx*, *pax1/9*, *slc25A21*, *mipol1*, and *foxA*) found complete only in Ambulacraria and Chordata. Several of these genes have functional links to pharynx patterning and to the formation of pharyngeal slits. Different protostomes do have some of these genes linked, i.e., *nkx2.1* *nkx2.2* (various protostomes), *nkx2.2 msxlx* (*Lottia*), *pax1/9 slc25A21* (*Lottia*), *mipol1 foxA* (various protostomes), and *pax1/9 foxA* (distant but on the same chromosome in *Caenorhabditis*) (*17*). *Msxlx* is adjacent to three *nkx2.1/2*-type genes in *Trichoplax* and is also relatively close to *foxA* in the genomes of medusozoan and octocoral cnidarians (cnidarians lack *pax1/9*). We have also identified a linkage between *msxlx* and *pax1/9* in the genome of the protostome *Phoronis australis* (separated by 0.34 mb, with 17 intervening genes) (Supplementary Text). These links raise the possibility that at least five (*nkx2.1*, *nkx2.2*, *msxlx*, *pax1/9*, and *slc25A21*) and perhaps all of the genes in the cluster were linked in the bilaterian common ancestor and that the cluster is a bilaterian character that has been dispersed in different protostome lineages (Fig. 5A).

## DISCUSSION

### Implications of difficulties resolving relationships between Protostomia, Chordata, and Xenambulacraria

We have shown that the branch leading to monophyletic Deuterostomia, if it exists, is short and weakly supported; for comparison, we demonstrate unequivocal strong support for Protostomia especially from higher-quality gene alignments. Comparisons of rates and relative branch lengths between datasets and the effects of model misspecification show that given some support for monophyletic Deuterostomia correlates with conditions expected to enhance systematic error. The contention that monophyletic Deuterostomia could result from systematic error is given some support by our simulation experiments. Systematic error is especially likely to affect our ability to reconstruct relationships between taxa separated by the very short branches we have described. Recent work has shown that Xenacoelomorpha are very likely to be the sister group of the Ambulacraria (*4*, *5*, *18*); nevertheless, experiments in which we removed the contentious Xenacoelomorpha did not change the results showing that this controversy should not affect our interpretation of the findings.

Such a short deuterostome branch has important consequences for our understanding of character evolution at the base of the Bilateria. It implies some combination of (i) a slow rate of change across many genes in the branch leading to the (hypothetical) deuterostome ancestor and (ii) a short period of time separating the last bilaterian common ancestor and the last deuterostome common ancestor (*4*, *19*). Hybridization between lineages following speciation is another process that could blur the distinction between these ancestral taxa. If the deuterostomes are paraphyletic, as some analyses suggest, then the last common ancestor of Chordata and Xenambulacraria was the last common ancestor of all Bilateria; Urbilateria would have had the characteristics that have, until now, been used to define deuterostomes—characters common to Xenambulacraria and Chordata would be bilaterian plesiomorphies.

The idea that Urbilateria had some deuterostome characters is not new. Grobben included the phylum Chaetognatha in his Deuterostomia (*20*). Chaetognaths are now known to be lophotrochozoan protostomes (*5*, *21*) but have the three main deuterostome characters (*22*) of radial cleavage, forming their coeloms/mesoderm by enterocoely and their anus forms in the vicinity of the closed blastopore—the mouth is a secondary opening—and by this definition, they are deuterostomes. Deuterostomy and radial cleavage are also found in some Ecdysozoa, as observed in priapulid worms (*23*, *24*). Radial cleavage, enterocoely, and deuterostomy are characters found in both protostomes and deuterostomes and are likely, therefore, to have been primitive characteristics of the Bilateria.

Members of both Chordata and Xenambulacraria also have pharyngeal slits, a postanal tail, and a form of endostyle (a pharyngeal tissue that secretes iodine-rich mucus for filter feeding) (*25*). If the deuterostomes are paraphyletic, then this would suggest that these were also characteristics of Urbilateria, implying that they have been altered beyond recognition or lost in the lineage leading to the protostomes. Our evidence that the pharyngeal cluster of genes likely existed in the protostome ancestor implies that stem protostomes may have had pharyngeal slits.

A number of fossil forms from the Cambrian period have been interpreted as stem deuterostomes because, while lacking most defining characteristics of extant deuterostome phyla, they have pharyngeal slits (Fig. 5B) (*26*–*29*). The pharyngeal slits in vetulicolians (*26*, *27*) have been used as evidence for placing them as stem- or total-group deuterostomes, with the bipartite vetulicolian body plan taken as a model for the ancestral deuterostome (*26*, *27*, *30*). If pharyngeal slits were present in Urbilateria, then this removes the key character supporting the placement of vetulicolians with deuterostomes, and the presence in vetulicolians of a terminal anus and segmentation (*27*) could indicate that they are stem protostomes that had lost a postanal tail. Banffiids, which lack pharyngeal slits but are otherwise morphologically similar to vetulicolians (*29*), might occupy a more derived position in the protostome stem group.

This suite of complex characters in Urbilateria suggests a relatively large, filter-feeding animal arguing against a small and simple urbilaterian (*2*, *31*). The apparent lack of Precambrian bilaterians would not be easily explained as the result of poor preservation potential due to small size and simple morphology, emphasizing the gap between molecular clock estimates for the origin of bilaterians and their oldest fossil evidence (*32*).

While we have shown that support for monophyletic Deuterostomia is exaggerated by systematic errors, we have not attempted definitively to resolve this polytomy. The conclusions we have reached with regard to character evolution are, however, unlikely to be affected by resolving the relationships between these extremely short branches, assuming a correlation between molecular and morphological evolution. Nevertheless, recent analyses using large datasets and the best available models give weak support to a sister group relationship between the Chordata and Protostomia to the exclusion of the Xenambulacraria.

To facilitate future studies of this problem, we propose the name “Centroneuralia” for a potential Chordata and Protostomia clade, recognizing their shared characteristic of a centralized nervous system. We propose the name “Orthozoa” for a potential Xenambulacraria and Prostostomia clade, recognizing the shared character of having an orthodox dorsoventral orientation when compared with the axis-inverted Chordata.

## MATERIALS AND METHODS

### Datasets used

All analyses were based on five recently published phylogenomic datasets (*2*–*6*) covering all major clades of the animal phylogeny. For convenience, we will refer to each dataset by the name of the first author of each study (i.e., “Marletaz,” “Philippe,” “Laumer,” “Cannon,” and “Rouse”). In all our analyses, we kept the full set of taxa apart from the fast-evolving Acoelomorpha species that have been shown to be prone to phylogenetic inference errors (*4*). In our analyses, the Cannon dataset consisted of 67 taxa and 881 genes (“proteinortho.phy” in the original study), the Laumer dataset consisted of 422 genes and 152 taxa (167 taxa in dataset M in the original study), the Marletaz dataset consisted of 70 species and 1174 genes (alignments provided in the original study after the HMMClean and BMGE filtering), the Philippe dataset consisted of 51 taxa and 1173 genes (trimmed alignments provided in the original study), and, last, the Rouse dataset consisted of 26 taxa and 1178 partitions (“70.fas” in the original study). Each dataset contained several representatives of the major metazoan clades, specifically the two Protostomia clades Lophotrochozoa and Ecdysozoa, the two Deuterostomia clades Chordates and Xenambulacraria, as well as several nonbilaterian phyla.

### Measuring lengths of branches leading to deuterostomes and protostomes

The protostomes and the deuterostomes are traditionally treated as two equally strongly supported clades. To assess whether molecular phylogenetics supports this perception of bilaterian evolution, we compared the branch lengths of the two clades across the five concatenated datasets as well as for each gene of each dataset individually. For each dataset, we assumed the tree topology as reported in the original studies, specifically, for Cannon, Laumer, Marletaz, and Rouse the trees we used relate to Fig. 2, Fig. 2A, Fig. 2, and Fig. 3 from their respective publications, while for the Philippe data, we assumed the relationships as in the “PHILIPPE-ALLSPP-NOACOEL-CATGTR-100JP.tre” from the original publication. For the Cannon, Laumer, and Rouse data, *Xenoturbella* are considered to be sister to Ambulacraria rather than Nephrozoa. This position for *Xenoturbella* in the absence of the long-branched Acoelomorpha is supported by these datasets. For the Philippe and Marletaz data, we altered the tree only to enforce DM, as this was not supported in the published phylogenies. For the analyses performed per gene, we cropped the original trees using newick-tools (*33*), such that only the taxa present in the relevant gene alignment were present. Given the fixed topology, the branch lengths were estimated with IQ-TREE (*34*) under the state frequency homogeneous model LG + F + G. We used the optimized topologies to measure the length of the branch leading from the common ancestor of Bilateria (protostomes and deuterostomes) to the common ancestors, respectively, of deuterostomes and protostomes.

### Measuring lnLikelihood support for the branches leading to deuterostomes and protostomes

We performed a second analysis where we measured the difference in the likelihood score between the fully resolved phylogeny and for the topology after collapsing either the protostome or the deuterostome branch into a polytomy.

Collapsed Protostomia branch = (Lophotrochozoa, Ecdysozoa, (Ambulacraria, Chordata))

Collapsed Deuterostomia branch = ((Lophotrochozoa, Ecdysozoa), Ambulacraria, Chordata)

For calculating the lnLikelihoods, we used the LG + F + G model. We measured the difference in lnLikelihood in both the five full concatenated alignments and for each gene from each dataset individually.

### Measuring difference in lnLikelihood per gene for topologies with monophyletic and paraphyletic deuterostomes and protostomes

We measured the proportion of genes in each dataset that strongly support the monophyly of the deuterostome clade DM over the two alternative paraphyletic topologies. Specifically, we compared the lnLikelihood of the topologies, assuming monophyletic deuterostomes DM and two possible paraphyletic alternatives.

Deuterostome paraphyly “D1”: (Chordata, (Xenambulacraria, (Lophotrochozoa, Ecdysozoa)))

Deuterostome paraphyly “D2”: (Xenambulacraria, (Chordata, (Lophotrochozoa, Ecdysozoa)))

We performed the equivalent analyses for the protostomes; specifically, we compared the lnLikelihood of the topologies assuming monophyletic protostomes PM and the two possible paraphyletic alternatives.

Protostome paraphyly “P1”: (Ecdysozoa, (Lophotrochozoa, (Xenambulacraria, Chordata)))

Protostome paraphyly “P2”: (Lophotrochozoa, (Ecdysozoa, (Xenambulacraria, Chordata)))

To achieve this in both cases, we calculated the log likelihoods for the three alternative topologies using IQ-TREE under the LG + F + G model. The analyses were performed twice, once with and once without *Xenoturbella.*

In all cases, the topologies were fixed as described earlier, and we manually adjusted them to produce the two alternative hypotheses rendering protostomes or deuterostomes paraphyletic. As before, each of the topologies was trimmed to the taxa present in each gene. We visualized the relative support of each gene for the three alternative topologies affecting either deuterostomes or protostomes using a method adapted from that described in (*35*). By scaling the three likelihoods in the range [0,1] such that lnLn1 + lnL2 + lnL3 = 1 (the relevant python script “likelihood_transform.py” is available at https://github.com/MaxTelford/MonoDeutData), we could use their transformed values as coordinates in a ternary plot, whose corners represent the three topologies. We considered a gene to support a particular topology if the corresponding scaled likelihood was larger than two-thirds (and the likelihoods for the other two topologies were therefore smaller than one-third). We divided the triangle into compartments to reflect these cutoffs and plotted the points using the R package “ggtern” (*36*) (the relevant R script “plot_triangles.R” is available at https://github.com/MaxTelford/MonoDeutData). Identical steps were taken when repeating these analyses with datasets from which the Xenacoelomorpha had been excluded.

### Comparing the phylogenetic informativeness of genes supporting topologies with monophyletic and paraphyletic protostomes and deuterostomes

To identify potential reasons for individual genes supporting alternative topologies for both the deuterostome and the protostome clades, we evaluated two parameters known to be related to errors in phylogenetic inference. For each gene alignment from each dataset, we first measured the alignment length. Subsequently, we inferred the maximum likelihood phylogeny for each gene with IQ-TREE under the LG + F + G model and measured the monophyly score of the tree [this is a measure of a dataset’s ability to reconstruct known monophyletic groups and is described in (*36*)]. We used Welch’s *t* test to determine whether the difference in these scores between the sets of genes supporting different topologies was significant (table S3).

### Measuring branch lengths of different clades across datasets

We assessed how the average branch lengths of the two clades Protostomia and Deuterostomia and of the outgroup clan differ across the five datasets. Using the complete alignments, we estimated the branch lengths under the LG + F + G model, assuming the DM topology with IQ-TREE. To measure the average tip-to-stem branch length in each clade or clan, we used the “pynt.py” script (available at https://github.com/MaxTelford/XenoCtenoSims). We additionally measured the tree length (sum of all branch lengths) for the five datasets after reducing each of them to the eight species they all had in common (i.e., *Amphimedon queenslandica*, *Nematostella vectensis*, *Strongylocentrotus purpuratus*, *Ciona intestinalis*, *Peripatopsis capensis*, *Priapulus caudatus*, *Capitella teleta*, and *Lottia gigantea*).

### Measuring the effects of different models on estimates of branch lengths

We compared the estimates for the deuterostome, protostome, and bilaterian stem branch lengths across four models accommodating or not rate and state compositional heterogeneity across sites. We performed this analysis only for the Laumer dataset that showed the strongest support for DM. To lessen the computational burden, we reduced the dataset to 36 taxa that cover all major branches of the phylogeny, selecting taxa with fewest missing data and randomly selected 50,000 of the original 106,186 alignment sites (“reduced-Laumer”). Using the reduced-Laumer dataset and assuming the DM topology, we performed a phylobayes-mpi [version 1.8 (*37*)] run for four substitution models, i.e., LG (homogeneous model in terms of state composition and rates across sites), LG + G [homogeneous state composition and site heterogeneous rates], C60 + LG + G [heterogeneous model assuming 60 different sets of amino acid state frequencies (*38*)], and the infinite mixture model CAT + LG + G model (*39*). We performed 10,000 Markov chain Monte Carlo (MCMC) cycles for all models except for the CAT + LG + G, for which we performed 20,000. For all four runs, we used the final 5000 posterior tree samples to calculate the average stem branch length of the deuterostomes, protostomes, and bilaterians.

### Cross validation to measure fit of different models

To compare how the branch length estimates relate to the degree of fit of each of the four models, we performed cross-validation analyses as described in the phylobayes-mpi manual. Using the reduced-Laumer dataset, we created 10 training datasets each consisting of 10,000 sites and 10 test datasets of 2000 sites each. We ran phylobayes-mpi for 3000 MCMC cycles for each of the training datasets and for each of the four models. Subsequently, using the “readpbmpi” program (distributed with the phylobayes-mpi) under the “-cv” option, we calculated the posterior mean cross-validation score with a burnin of 2000 and sampling a frequency of 0.1. As expected, less complex models have significantly worse fit to the data than the more complex models (average cross validation scores: LG, −74,877.78; LG + G, −70,348.95; C60 + LG + G, −68,707.89; CAT + LG + G, −68,031.36).

### Simulations: Systematic error

We simulated sequence alignments using parameters that match those measured from empirical sequences under the three topological hypotheses relating the deuterostome clades. As before, we based these analyses on the reduced-Laumer dataset. The parameter estimates and simulations were carried out with phylobayes-mpi. Identical steps were taken when repeating these analyses with a dataset from which *Xenoturbella* had been excluded.

The two steps were as follows:

1) We estimated the posteriors of branch lengths and model parameters using the CAT + LG + G model on three alternative fixed topologies relating the deuterostome clades. For each fixed topology, we performed 20,000 MCMC cycles with a sampling frequency of 1.

2) Using the final 5000 posterior samples, we subsampled with a frequency of 1 in 50, which gave us a subset of 100 posterior samples. Using these combinations of branch lengths and model parameters, we simulated data with the “readpb_mpi” tool under the “ppred” option.

For phylobayes runs using all three fixed topologies, the effective sampling size (ESS), for some of the model parameters, was lower than 100, but in all cases >50, possibly suggesting inadequate MCMC mixing and lack of convergence. This is a known problem of phylobayes when using large datasets. To ascertain whether the results from our simulations were affected by inadequate mixing (or by a sampling effect), we repeated the procedure for a smaller fraction (10,000 randomly selected sites) of the Laumer alignment. In this case, we performed 10,000 MCMC samples, and the ESS values were above 100 for all parameters with a 25% burnin. The results of the simulations based on this dataset were in broad agreement with the results based on the larger dataset (table S4).

For each of the simulated datasets (simulated under the best-fitting state-frequency heterogeneous CAT model), we performed two phylogenetic inference analyses using IQ-TREE: (i) under the state-frequency homogeneous model LG + F + G and (ii) the state frequency heterogeneous model C60 + LG + F + G under the PMSF (posterior mean site frequency) approximation (*40*). The C60 model implements an approximation of the CAT model of Phylobayes but with 60 precomputed site frequency categories (*5*) and is considerably faster to run in a maximum likelihood framework (*40*), enabling the analysis of multiple simulated datasets. We wanted to test the possible influence of an LBA attracting long-branched protostomes to the long branch leading to the outgroup resulting in artifactual support for monophyletic deuterostomes. We repeated the previous analyses after removing the longest protostome branches (i.e., *Diuronotus aspetos*, *Geocentrophora applanata*, *Schmidtea mediterranea*, *Echinococcus multilocularis*, *Adineta vaga*, *Loa loa*, *Caenorhabditis elegans*, and *Hypsibius dujardini*) and the outgroup taxa (i.e., *Polypodium hydriforme*, *A. queenslandica*, *Beroe abyssicola*, *Mnemiopsis leidyi*, and *Salpingoeca rosetta*). Last, we performed an equivalent experiment by removing the 13 shortest protostomes (*C. teleta*, *Helobdella robusta*, *Phyllochaetopterus* sp., *Daphnia pulex*, *Limulus polyphemus*, *Hemithiris psittacea*, *Membranipora membranacea*, *L. gigantea*, *Neomenia* sp., *Phoronis psammophila*, and *P. caudatus*) and outgroup taxa (*Craspedacusta sowerbyi* and *N. vectensis*) and inferred the tree topologies under the LG + F + G model. In all cases, we measured the proportion of simulated datasets supporting each of the three potential topologies.

### Gene order

Gene order was determined from the National Center for Biotechnology Information (NCBI) genome gff files, or gff files distributed with sequence data from the Marine Genomics Unit of the Okinawa Institute of Science and Technology (https://marinegenomics.oist.jp/), or other online resources related to original publications. Orthologs of selected genes were determined from phylogenies of corresponding Pfam domains (e.g., Homeobox, Forkhead, etc.). Briefly, Pfam hidden Markov models were searched against a metazoan protein database (hmmsearch), and sequences were aligned (hmmalign) and then trimmed (esl-alimask and esl-alimanip) on the basis of posterior probabilities of correct alignments and length of remaining sequence. Phylogenies were constructed using IQ-TREE, allowing the optimum model to be selected from the LG set (i.e., “-mset LG” in IQ-TREE). Orthologs were identified by inspection of the phylogenetic tree around the relevant gene (e.g., *msxlx*, *pax1/9*, etc.).

### Testing the phylogenetic distribution of supposed deuterostome-specific genes

Deuterostome novelties of Simakov *et al.* (*16*) were searched against selected protostome and nonbilaterian metazoan datasets. Most gene sets (*Lingula*, *Priapulus*, etc.) were obtained from the NCBI genomes resource (accessible at www.ncbi.nlm.nih.gov/genome/browse#!/overview/), except *Phoronis australis* (OIST; see above). Sponge transcriptomes were assembled from reads retrieved from the European Nucleotide Archive:

*Plakina jani* (*41*): SRR3417194_1.fastq.gz and SRR3417194_2.fastq.gz.

*Corticium candelabrum* (*39*): SRR499817_1.fastq.gz, SRR499817_2.fastq.gz, SRR499820_1.fastq.gz, SRR499820_2.fastq.gz, SRR504694_1.fastq.gz, and SRR504694_2.fastq.gz.

Sequences were processed into “left” and “right” read files adding “/1” and “/2,” respectively, to identifiers (e.g., for a “_1.fastq” file: perl -pe ´s/^@(SRR\S+)/\@$1\/1/´) and then assembled using Trinity-v2.8.4 (*42*) with default parameters: trinityrnaseq-Trinity-v2.8.4/Trinity --seqType fq --max_memory 128G --left fastq/left.fastq --right fastq/right.fastq --CPU 32.

*Oscarella carmela* proteins were downloaded from compagen (www.compagen.org/datasets.html; file OCAR_T-PEP_130911) and *P. australis* from OIST (see above). Candidate orthologs were identified via reciprocal best hits and phylogenetic analysis of relevant Pfam domains, as described above (section “Gene order”). Sequences were also searched against the NR database of the NCBI to test likely monophyly of metazoan (in this analysis, generally, sponge and bilaterian) proteins based on clear separation of bit scores, using blastp (v2.10.0+) (*37*).

## Supplementary Material

http://advances.sciencemag.org/cgi/content/full/7/12/eabe2741/DC1

Adobe PDF - abe2741_SM.pdf

Lack of support for Deuterostomia prompts reinterpretation of the first Bilateria

## SUPPLEMENTARY MATERIALS

Supplementary material for this article is available at http://advances.sciencemag.org/cgi/content/full/7/12/eabe2741/DC1
