# Supplementary material for: Lack of support for Deuterostomia prompts reinterpretation of the first Bilateria
Source: Sci Adv. 2021 Mar 19;7(12):eabe2741. doi: 10.1126/sciadv.abe2741 (PMC7978419; doi:10.1126/sciadv.abe2741)
Supplement: http://advances.sciencemag.org/cgi/content/full/7/12/eabe2741/DC1 [file supp_7_12_eabe2741__7.12.eabe2741.DC1.html]

Science Advances | Science AdvancesAAASSearchScience AdvancesMenu

## Supplementary Materials

# Lack of support for Deuterostomia prompts reinterpretation of the first Bilateria

Paschalia Kapli, Paschalis Natsidis, Daniel J. Leite, Maximilian Fursman, Nadia Jeffrie, Imran A. Rahman, Hervé Philippe, Richard R. Copley, Maximilian J. Telford

Download Supplement

**This PDF file includes:**

- Supplementary Text
- Figs. S1 and S2
- Tables S1 to S4
- References

**Files in this Data Supplement:**

- Adobe PDF - abe2741\_SM.pdf
