# Supplementary material for: Lack of support for Deuterostomia prompts reinterpretation of the first Bilateria
Source: Sci Adv. 2021 Mar 19;7(12):eabe2741. doi: 10.1126/sciadv.abe2741 (PMC7978419; doi:10.1126/sciadv.abe2741)
Supplement: http://advances.sciencemag.org/cgi/content/full/7/12/eabe2741/DC1 [file supp_7_12_eabe2741__abe2741_SM.pdf]

Science Advances
AAAS

advances.sciencemag.org/cgi/content/full/7/12/eabe2741/DC1

Supplementary Materials for

Lack of support for Deuterostomia prompts reinterpretation of the first Bilateria

Paschalia Kapli, Paschalis Natsidis, Daniel J. Leite, Maximilian Fursman, Nadia Jeffrie, Imran A. Rahman, Hervé Philippe, Richard R. Copley, Maximilian J. Telford*

*Corresponding author. Email: m.telford@ucl.ac.uk

Published 19 March 2021, *Sci. Adv.* **7**, eabe2741 (2021)
DOI: 10.1126/sciadv.abe2741

This PDF file includes:

Supplementary Text
Figs. S1 and S2
Tables S1 to S4
References

Supplementary text: Results on deuterostome novelties

Pharyngeal gene cluster

Linkage of nkx2-1/2 to msxlx in Trichoplax:

On scaffold NW_002060943.1, NCBI *Trichoplax* genes, XP_002107829.1, XP_002107830.1, XP_002107831.1 encoding nkx2-1/2 like homeoboxes are adjacent to likely msxlx ortholog XP_002108628.1.

>Ta|XP_002107829.1 hypothetical protein TRIADDRAFT_9507, partial [Trichoplax adhaerens]
KRRILFTQMQVSELEKRFKRQRYLSAQEREHLAKLIDLTPTQVKIWFQNHRYKCKRQF
>Ta|XP_002107830.1 hypothetical protein TRIADDRAFT_9561, partial [Trichoplax adhaerens]
KKKRRILFTKNQTDQLEKRFRQQKYLSAPEREHLANMIKLTPTQVKIWFQNHRYKCKRQ
>Ta|XP_002107831.1 hypothetical protein TRIADDRAFT_9531, partial [Trichoplax adhaerens]
RRRILFSSQQVEVLQNRFQINIYLSPIEREQLARKLHLSPKQVKVWFQNQRYKKRKK
>Ta|XP_002108628.1 hypothetical protein TRIADDRAFT_51739 [Trichoplax adhaerens]
MVVAKREKVKNNKKSNAKCHRLGQSCPTLTEKPKDEKNFQTDFSKEPIANKFTAPTTMPF
SVDRILGFANSTVAPMAIDPNYAYYRNSYNPNDFRYTYPAPYFGLGRLDWLQTNPHFTPV
TVKNDNSNEDGQKNCTQKKRRISRCPRIPFSKDQLNLMEDVFQQQQYLSPRDIENLCRKL
DLKEHRVKNWFQNRRAREKRARTSSSITVLTNQYETSPDSKKNVNSNETVTPTANPMYLT
YTPNSPSKNALQDNRWLVTIPEKIQNIDQHKD

Linkage of nkx2-1/2 to msxlx and foxA in the octocoral Xenia sp.:

The octocoral *Xenia sp.* encodes likely orthologs of several pharyngeal cluster genes on the same scaffold, within around 100 genes (0.7Mb) of each other for the core transcription factors:

HiC_scaffold_25 5648642 Xe_021979 # pharyngeal mipol-like
HiC_scaffold_25 5749435 Xe_022000 Forkhead:HNF_C # pharyngeal foxa-like
HiC_scaffold_25 5760896 Xe_022001 Forkhead:HNF_C # pharyngeal foxa-like
HiC_scaffold_25 6073776 Xe_022051 Homeobox # pharyngeal nk2-like
HiC_scaffold_25 6459615 Xe_022099 Homeobox # pharyngeal msxlx-like

>Xe_022000-T1 Xe_022000
MMDSLLTSDYLQDPLDPKARQVKDKEKSMTSRRNFTHAKPPYSYISLITMAIQQSPNKML
TLSEIYQFIMDLFPYYRANQQRWQNSIRHSLSFNDCFVKVPRSPDRPGKGSYWTLHPDCG
NMFENGCYLRRQKRFKADKKPNLNHLVKPGPYVPGVPKVQGGKTFATPSFLAPTHYGGFR
PGFNHPFAIKNIIADHEMDFRGYDSMHFNPYHSGSLSTPPPVPAMSPMSSFGLPKTTLDP
PSLMTSDANLTPYYPSCGYINTSSSCSLSGYT
>Xe_022051-T1 Xe_022051
MSNSANGVHSIENILETCERKKLSQQLTTTRKIQCFKPVLSSVQSIAEPCGLPNDKDVSK
EISFDYSIESGVQKKRTKNNEINTFTVRTPSHINTEQTSHIEIEKNTSSRKKTKAKKRVL
FTRAQVFELERRFRVQKYLSAVEREQLAKVTNLTPTQIKVWYQNHRYKNKKQNIRETAET
EKLWDFYEYGRNRQLINQMFQNMNSTSSSATLNNISHDNMQ
>Xe_022099-T1 Xe_022099
MYLKIQAESFLIILYTLQEKFNWILKRFAIQPQPFWLRTVGRKKTQYEEKNKAILQPFHT
LHNSPREESSKRRPKQKRKPGRNPRVPFTPKQLTTLENKFETMKYLTSDEVRSLCLVLTL
PENKIKIWFQNRRAREKRKSSQSSDEYVDVMTIDGENS

(Data downloaded from: http://cmo.carnegiescience.edu/data/)

Linkage of msxlx and pax1/9 in Phoronis australis:

Phylogenetic analysis identified g118.t1 as msxlx and g100.t1 as pax1/9, both encoded on 'scaffold1' (annotation file: 51_pau_v2.gff). There are 17 intervening genes but 3 of these have no database hits in the NCBI NR protein database.

```
>Pa|g100.t1
MDQAFGEVNQLGGVFVNGRPLPNAIRLRIVEMAQLGVRPCDISRQLRVSHGCVSKILARY
HETGSILPGAIGGSKPRVTTPNVVKHIKMYKERDPGIFAWEIRDKLLSDGVCDKFNVPSV
SSISRILRNKLGSIQQQQFDPYKDSKPLYNLYPYSCASGGPPLSPGNNLVSQPQNVNMPP
MAKPTNHPSAVMRWPSSHAMNNILGLRQSCNQAQENSCMLNNPYKHHDSGQYNYYHMPST
VPSMYLQS
>Pa|g118.t1
MALLNSLYGPGKYPAFYMSHGNYKNFYANLVQGLGGMPPTNYDLTRVQLARKPEMAAATS
YQLRIPSPGQRKPSSPRATSTGVVESEKHSAETVDKNLIKPSQNPLTNFSIEGILGRKET
VTSSESEDAYDDDEEENEGITDKDDGIHNISRDSNVESESVNLDRQNSLEKDNIDETEFS
WLQCTRYKPPKLPRSRKREGVKKRKLGRNPRVPFTQQQVGILEQKFRQTHYLSSVDVLEL
STALNLTETRVKIWFQNRRARERRDRECKKTNPHVRPPQNASSAAGSSLSPYHGAIPWPL
PLPPNPTTLAQFSQALQYGPHSAFSPVPFIYTSQPHLAASPFTFYRVPEAKE
```

(Data downloaded from: https://marinegenomics.oist.jp/pau_v2/viewer/download?project_id=51)

We find no evidence for pax1/9 orthologs outside of the Bilateria (in the context of our analysis, cnidarians or *Trichoplax*).

Gene novelties

We examined the 31 genes / gene families highlighted as deuterostome novelties in the supplementary information of Simakov et al. to test their status in light of new sequence resources. Our primary consideration has been the identification of candidate orthologs within protostome and non-bilaterian animal sequence sets. Identification of such sequences leads to the inference that the gene was present in the common ancestor of protostomes and deuterostomes (i.e. Urbilateria). Simakov et al. classify some genes as deuterostome specific, despite having non-bilaterian animal representatives, as a consequence of inferred loss within protostomes. Such genes are plesiomorphic with respect to Bilateria and therefore unsuitable as markers of deuterostome monophyly. We recognise that demonstrating deuterostome monophyly was not the purpose of Simakov et al. in gathering these data.

In the "Novel aspects of TGFbeta signaling genes in deuterostomes" category of Simakov et al, there is good evidence of urbilaterian ancestry for 3 out of 5 of their examples, with a potential fourth dependent on the precise timing of a gene duplication event (for which no evidence is available). In their "Sialo-glycoproteins and the evolution of muco-ciliary filter feeding" category, out of 7 examples, we consider 6 likely present in Urbilateria. In the category "Deuterostome novelties for physiological-metabolic specializations" at least 10 out of 14 examples were likely present in Urbilateria with a further example likely hemichordate, rather

than deuterostome, specific. Finally of the 5 examples in "Deuterostome novelties without eukaryotic or prokaryotic homologs", we identify one that was likely present in Urbilateria.
s
We review our reasoning below. Homoscleromorph (*Plakina*, *Corticium*) sponge transcriptomes were assembled from reads using the methods described, and we provide example sequences from these assemblies where relevant. We report bit scores as these do not vary with database size (scores from blastp, see methods). All hits were statistically significant ($P < 0.001$) unless otherwise noted. Section headings refer to human gene names, with multiple paralogs indicated in parentheses. Genes are presented with the same section numbering as Simakov et al.

Species abbreviations:

Sponges: P_jan *Plakina jani*, C_can *Corticium candelabrum*, O_car *Oscarella*, Aq *Amphimedon queenslandica*
Cnidarians: Ep *Exaiptasia pallida*, Of *Orbicella faveolata*, Pd *Pocillopora damicornis*
Protostomes: La *Lingula anatina*, Pa *Phoronis australis*, Pc *Priapulus caudatus*
Sequences can be retrieved via the accessions at the NCBI, excepting the homoscleromorph sponges.

Novel aspects of TGFbeta signaling genes in deuterostomes

1. LEFTY (1,2)

The LEFTY protein is composed of a pro-peptide and TGF-β signalling domain. Simakov et al. reported the existence of a lophotrochozoan ortholog of the LEFTY pro-peptide lacking the signalling domain (and we thus infer its presence in Urbilateria). In agreement with them we were unable to identify protostome or non-bilaterian LEFTY signalling domain orthologs.

Our assessment: deuterostome specific (signalling domain only).

2. GDF1/Univin

Blast searches and phylogenetic analysis revealed an ortholog in the protostome brachiopod *Lingula anatina* (in a similar tandem duplication arrangement with BMP2/4 to that discussed by Simakov *et al.*). The presence in *Lingula* has also been noted by Luo and co-workers *(43)*.

Our assessment: present in Urbilateria, because present in protostomes and deuterostomes.

Example sequence:

```
>La|919024921 ref|XP_013396302.1| PREDICTED: univin-like [Lingula anatina]
MQLPDSPGGGVCYLFLFMAVRLSSTAVGLQTAIKMHQGAILRGHTTVDGEEHLETAAHLV
QSDISHSRSQHDELKMLKLFGLSERPSPKPNSTVPGFMYQLYEKVRLSEERDQARCHFSD
QSIPGNVIRSLRNKGPPKDQDLRFHPNGVRIFFNLSVVPALESVTRAELRIESSQLTKQD
RAENSQKCKISIYQIRRTLNIRISSSIDSLHLKHISSSVFELNTENDSLDSWQSPDLIEI
VKFWSENPTKNHGLYLLHEPLESNSVENRDSLCSNLESSSISLLVVTSDTSKCHHRVRRS
KPTINFARSNLCRRHALFIDFKDVGWAKWIISPVVYQAYRCQGDCPFPLNEHLNGTNHAI
IQNLVNSMYPSTVPRACCAPTHLSAMSMLYFDNHDNVVLRKYEDMIVDSCGCH
```

3. TGFB (1,2,3)

The discussion of Simakov et al. notes the presence of sponge and anthozoan sequences and that deuterostome TGFb2 genes were “likely derived from a bilaterian ancestral sequence”. In agreement with them, we identified orthologs of TGFB1/2/3 in anthozoans and sponges.

Our assessment: present in Urbliateria, because present in deuterostomes and non-bilaterian animals.

Example sequences:

```
>Ep|XP_020915263.1 transforming growth factor beta-2-like [Exaiptasia pallida]
MFLISCFSKALLLLAVCQDLGMFFVNGKSRLKESRIQAVRGQILTKLGLTSPPDEGARVR
PKREELEAFKAVRNEAFQHQNVDENCIRNDKSASYFAKSIVSLHLVQRTGKTIQYEDPAK
KRVHIVDINTLTFDATQLDGEHHIEKAEMRIDPNYFPTIQGTTIKACMVDRLSSSSIHNT
CSQVLDSKFIDSQADINLDVTDAVKKRXMAPKSMAFVKVIQXGMEQNGYDSSSTLSWEPY
VLVWYIPMNRYTIERRRRHRRSLDLAFCNQRPTEKRCCLRSLYIDFQKDLKWNWIHAPLG
FHANYCKGTCPFLWGSSNQTHHTSIMALYNSINPNAPSEPCCVASSYKPLVILHYVKGQP
KIEQLNNMVVTSCTCL
>Of|XP_020607064.1 transforming growth factor beta-1-like [Orbicella
faveolata]
MSLLSSQMLGTFPLYVWFLIWMNVVGVAFITGCKTCNSRQAREKRLIAVQGQILAKLGLT
KPPNGEEIFMNVSREVMQTYKSAVQEKDKLLLESKLCRSQVDADEEYFAKRVERLLLEKE
VARTVVPSKYAKLRLSTCPKGKNDKDVYSLIFNLSKLVTESARVSLAELRLYQMPNQRQP
LSNVRIDVCENTGEHDDNAKNGCITALDSAWSLSSEEKWISFDITSVVQKWLEDSSTNYG
LTVTVTSSVPADKDERVSPIYLGGPAPKQMHDEDEEPWPNILVWFIPRERVHSTRRRNKR
SLDSNYCKKRSTETRCCLRSLYIDFKKDLRWKWLHAPKGFYANYCAGHCPLMWGVEKQNH
HTTIMSLYNKINPDAPGDPCCVPKTYEPLVVLYFKDGEPKIDELSNMAVSECTCL
```

4. THBS (1,2)

Simakov et al. note the presence of a sponge sequence with similarity to the N-terminal half of THBS1/2 and note that “it may reflect a metazoan sequence that persisted through the bilaterian ancestor to the deuterostome stem”. We agree with this, and identified further sponge genes including VWc, TSP1, TSP3 and repeats and other relevant domains.

Our assessment: present in Urbilateria, because present in deuterostomes and non-bilaterian animals.

Example sequence:

```
>P_jan|DN485_c0_g1_i9.p1
MIGDGEISTSRATLAALLGFAAVLFAFLTGTVDAESSPLEELNVLEEAGLSPSIALQTPR
VAYELHAGMPPVYHLDAPDKVFRVNSEQTERFATAVEKNKGFTFVANIQPAHGNNKGILV
SLDEPMSYRPAYLRLCYDGRMRKFLIEYSTKGNNVMTWFRDVHLSTQQMNRIVLVVHHLN
VMLYVNCRFVATAKLQGPIHATPLSERAEFRLGRTTAKIWSQPPYKGGIQEMKFLFKDMS
PEVLGSSCSIEEKTTYLKGLVAMNGCEYCPFVSQLVDVFQAMSTHLDDLKLRLQLRVRTN
PASKDTPTTEEGSCRDSRGYFYYHSETWAPSPCRNCSCYRGNITCLDTVCPKMDCDIFYQ
LPGQCCPSCWSALREPLPDDFVDHNNDINNRADVAVTWSDWGIWGLCSKSCGGGKQKRMR
```

```
NCNKITGYPGGHDCLGQMLETRVCNTYYCPIDGGWSNWTDWSPCSVSCEVGTQVRARRCD
NPVPAYDGVSCIGDDRETRACVLLEKCPTPCSPNPCFKGIVNCTEHGIKYQCGPCPVGYQ
GDGIHCRDLNECKLVPEACHPLASCKNKRPGYACSSCPAGYRGNRLKGIGLEDARTKRQV
CKDIDECAKNNGGCRHSPCINTIGSYHCGPCDPGYSREGRADGQCILVNVCPLGWHNCTG
NFKCQPAGNLSFVCVCPEIGHFFKDGMCIRDTDIDGYPDSAIDGCVEKWCKKDNCPTVPN
SGQEDHDNDGLGDACDDDDDNDGIKDSKDNCPLLRNVKQSDLDRDGVGDLCDNCVRTRNP
YQQDFDHDGLGNACDPDPDQDGIFTGVDNCPLARNEEQEDFDHDHVGDNCDNCPYDVNTN
QGDRDFDMVGDVCDTDIDSDGDGIQDDLDICRYQRNADQANHDSDAHGDLCDSDDDDDGF
PDNQDNCPLVKNSKQEDINGNGIGDLCENDYDGDSFANDIDVCPYNREIDRVDFLMFDQV
ALRYRKEPWWRVLANGAEIIQERVSSAGLAVGYHKFSNVDFNGTFFVNTDSRDDYFGFVF
SYQSNKRFYVVMWKKSKENYPSSKVSTALPGIQIKVVNSASGPSTALERALWHTGTTPRQ
VRLLWHDPKQIPWRSHTAYRWMLTHNAENGRIRLRIYDAGEEIIDTGYLVDRTLRGGRLG
FFALQQSQVFWSDLSYRCSGNVTDVDECSSILI
```

5. TGFBR2

TGFBR2 orthologs are present in Ambulacraria and Chordata. These are paralogous to Activin receptors. The TGFRB2 ectodomain sequence is similar to the extracellular domain of Actvin receptors. Although this is not statistically significant, neither is the similarity between, for instance the human TGFRB2 ectodomain and that of *Saccoglossus* or *Strongylocentrotus* orthologs, with a blast search of the human TGFRB2 ectodomain returning no significant non-chordate hits.

Our assessment: similar sequences were clearly present in Urbilateria. Deuterostome specificity depends on the precise timing of a gene duplication event, with deuterostome specificity (if this occurred on the deuterostome stem) or protostome loss (if origin on bilaterian stem).

Sialo-glycoproteins and the evolution of muco-ciliary filter feeding

6. GNE

We identified homoscleromorph sponge orthologs of GNE, with the best hit of human GNE to our sponge sequence database scoring 863 bits, and the best hit to bacteria scoring 318 bits.

Our assessment: present in Urbilateria, because present in deuterostomes and non-bilaterian animals.

Example sequence:

```
>C_can|DN12602_c0_g1_i3.p1
MDANNVQQRRQIKVCIATCNRADYSKLEPVMAGIQADPDMELSVIVLGSHLIDDYGSTHR
FISADGFSITQKLHTIVRGEDEAAMVESVGLSLVKLPDVLYRLDPDLLVVHGDRFDALAL
ATSAALMNYRILHLEGGEVSGTIDDSIRHAITKLAHYHACCTERARQRLIAMCESPSRVL
LSGCPSYDKLLRADTSSLERVCHRYQVDPNDFLIVIQHPVTTNQVESQKMFSVMVDAVIE
FNHSTLFLFPNVDAGSKDLTRIMRRRGLESHGQHPNISTIKHIPFDEFMILLSKCKCIVG
NSSAGVREAGAFGTPVVNVGSRQTGRETGENVVHVRDADSKEKVLRALQLQYGKRYPPSH
IYGDGRCIPRILQFIKEIPFKSGIQKGFNFPAMPASSSQDIDHILEIQSALAIDMGGTNL
RVAIVDLRGQIVYYTKVPNPPVFEERIDVLAALISDAKKKAVEHDCRLLGIGISTGGRVD
PKEGALLDATSLIHDWSYVNLRALLSSKVELPVWVDNDGNCAAQGERRFGKGQGVKDCIT
VIAGTGIGGGIILNGEVQYGSTFCAAELGHVVVSMDGPLCSCGQHGCVEAYAGGATLDRE
```

```
AKSLHEAGELNDFLEADREVSGVHLTAAAKLGNVNAEKIVRRAGTALGTAITSMLHVVNP
SHVILCGHLAPVYYNYVKAVIQQRALPSARKVEIVVSEISDPALLGAASLVLDYSIRRIY
GNSH
```

7. CMAH

We identified homoscleromorph sponge orthologs of CMAH, with the best hit of mouse CMAH to our sponge sequence database scoring 596 bits, and the best hit to bacteria scoring 219 bits. (Note this gene is absent in human).

Our assessment: present in Urbilateria, because present in deuterostomes and non-bilaterian animals.

Example sequence:

```
>P_jan|DN16311_c0_g1_i2.p1
MTASGSDRSLLLTLSKDIVDTLKPGVTIQTLNKHGILLYRSKGEDLKLRACKNVCHHQEG
TFIKDIESLTADACSSVVRCTKHGWKLDMATMEYINPPDCFTQEELLVEEDGKGNINLYQ
RIRPQQPWEKDGRPAQTIHAGEVKITYFTHACVEMNFDGTIFFTDPWLTGPAFARGWWLM
HEPPADWLDRLSKASLIYISHLHPDHLSYPTLKMLYQRNPHIPIYVGNTATLPFSRQHCY
GDEAYNVIVCELATWQEVSEDLRFMIMHDGIHTEMDTCLLIDYKGHLIMNTVDCSRPNDN
MLPKDVDLLMSDFAGGATGYPVSFYGGCYAEEWKRNFIQSERKKEINRKVAMAKKVNPQM
YLPFAGYFTEAHPSDEYIKERNRKNDPAVLNALLNKYAPNVQTWTPKPGAWVDLATLEVT
DPPIETPVFKTQWNFDLYTKLIDENIKFVPFQHPDSIHFYFQWVGLKDYNLVLRVIETDE
EFEKDPNGFDVLVDLQDVSFPSERPTRQHNYIEMKARIGVFRETMKFGYLWDNLYLGFQC
RLTREPDVFHFNFWNHMQLLLPSTPPDWAKFWKSKGGRVEELNGPRYMLPDLLPN
```

8. ST6GALNAC (3,4,5,6):

We identified homoscleromorph sponge orthologs of ST6GALNACs, with the best hit of human ST6GALNAC4 to our sponge sequence database scoring 214 bits, and the best hit to bacteria scoring 59.3 bits.

Our assessment: present in Urbilateria, because present in deuterostomes and non-bilaterian animals.

Example sequences:

```
>P_jan|DN13518_c0_g4_i1.p1
KARIMLMLLRLRPALALFLFLFAGIGIFIQWLRTRGDGVHDSKASLLTISESTSSQLQRD
LEVGVRVTTQISNRQTEAHDPDINHNNPSIGDPQSTSLEQRVPDTGVKEAKLTNSLKAQT
QANAEETKSDNAVHQLLSPNAVAPVPAEGILHDKLTVLPGYISVLDDQPLKLNCKTCALV
SSSGRILGKKKGSEIDQADCVFRMNSAPVEGFETDVGHKTTVRLLSQFSVPHGLRLVSHH
TDQPLNFFIAWGAEGHLGKKTQKYGMMLETAKKYPAMGIYRSSDKHYWYQDEVFQAETGQ
ERMKSGTWLSTGWFTFDVIKQACPRTKIYGMIPETFCRNGSAPPALYHYWERNAGDECSY
YRQMENRVRAAHRFMSEKTVFARWAIKYNMTFHVPDWDPKDYSHSEIKFPTFLS*
>O_car|m.311493
MLNRRISIALVALFGAVVLLWKLNSSMGNTQIVDNDNLLENLQVSTGLEETMTNLKKATE
RPVVVHASHSSASTEIKVASTNSGYPMMLNGYRSIIDPNEPLKMNCTTCSLVSSSGRILG
QSKGAEIDGADCVLRMNIAPVKGYEGDVGKRTTARILSQFSVKFARNQLLAAENLRYFIA
```

```
WGGNVHLGQKTANYKKMLEKAIQLPNIGFFRSSEEYYWYQDKVFENITGRPRQKSGTWLS
TGWFTFDVIKNACKRTKVYGMIPEVFCRDPTAPKALYHYWDPNAGDECAYYSRMENMRAG
AHRFMTEKAIFARWALQHSITFHSPDWDPKLHTEAHHKF*
>C_can|DN7762_c0_g2_i1.p1
MSRTMTYRLFRPRPIVALGLFVLAGSGILIQWLRFRSPSEGEKAPKSSEDELPQSLRQNI
GSDGGKRTENDELQEKKTTTENRVLHTAGQNSLVNQNGKTPDMDVVIDSKPAESEKSQNA
DLESEERESLETRDRPAVLNGYMSVLDDKPLTLNCKSCALVSSSGRVLGQKKGSEIDEAD
CVFRMNAAPAKGFEIDVGHKTTIRVLSQFSVAAGITQIIRGQESLSFFISWGADKHLGKT
TSKYREMVSTANKYPQIGFYRSSNEHYWYQDEIFEKETGQKRMSSGSWLTTGWFTFDVMK
QACPTTKIYGMVPEDFCKNPKAVPALYHYWEPRGGMECSYYAKMENNRRGAHRFMSEKTI
FARWAVKHNMTFHSPDWNPNDYIKKDIKFPMP
```

9. ST3GAL (1,2):

We identified homoscleromorph sponge orthologs of ST3GALs (in common with Petit et al., 2015 *(44)* and Simakov et al., 2017 *(16)*), although with representatives from multiple independently sequenced species. The best hit of human ST3GAL1 to our sponge sequence database scored 236 bits, and the best hit to bacteria scored 71.6 bits. Sponge sequences searched against the NCBI NR database retrieve vertebrates as best hits, suggesting all animal sequences form a clade.

Our assessment: present in Urbilateria, because present in deuterostomes and non-bilaterian animals.

Example sequences:

```
>O_car|m.10828
FAPESWNRRTFRFLCMVHKIMPRCRFVSFSIFLFVIMAIFVFIRHQTSWEESVSLEDPMM
RSQPAVKPEETKDQPAGKPKETKDQPAGKAEEAEDYPRRDSTICPNIGTLLANPNLPRIL
KSKFHPDVKVMLTQDTKTLDEPINSWWKKLQPFDNKRDFTELAEDLFTVIPGENPFSPGV
CRRCAVVGTAGRLKGARQGKLIDSFDIVIRMNRSPVKGYEVDVGSKTSYHLVYPESAVGY
RGAESSGKLVLFPFKVLDIEWLKSIFTTHPISKGWTHLPTNLGLKPTDAMVIHPEFIYYV
AKTWLEGKGRWASAGALSVVWALHICNEVDVFGFGANKYGNWDHYYEKFSSKEKDPFRRT
GVHDANIEETVRMELHKEKIIRFHPGNPA*
>C_can|DN3691_c0_g1_i6.p1
MNNAPVKGYEKDVGVKTTHHIVYPESARFYRGFRSSGKLVLLPFKLLDIEWLISTLTTHH
ISRGWTAVPTHLDLTGKDAMVIHPEFLYYARHTWYESQGRWPSAGALAIVWAIHICDEVD
VFGFGRNRFGNFHHYYEKVERTGKDPFRQTGVHNADIEEQVRAALDKGGAIRFHPGT*
>P_jan|DN4107_c0_g1_i3.p1
MNTAPVRGYEKDIGSKTTHHIVYPESAKNYRGAQSSGKLVLMPFKLLDIQWLISIFTTHN
ISRGWTSVPTRLALTGKDAMVVHPEFIYYVRHTWYESRGRWASAGGFAIIWALHICDEVD
VFGFGRNQYGNWAHYYDKFVSTGTDPFRRTGVHDADREEVVRLALTKAAVIQFHAGK*
```

10. ST8SIA (1,2,3,4,5,6)

We could not identify clear non-deuterostome equivalents of these genes.

Our assessment: subfamily specific to deuterostomes.

11. B4GALNT (1,2)

We identified likely orthologs of B4GALNTs in homoscleromorph sponges, with the best hit of human B4GALNT1 to our sponge sequence database scoring 213 bits, and the best hit to bacteria scoring 118 bits.

Our assessment: present in Urbilateria, because present in deuterostomes and non-bilaterian animals.

Example sequences:

```
>P_jan|DN3489_c0_g2_i4.p1
MARRPIVVRKYWRTVFGVTCITLLFWVSFRLVSLLRSYMVSLDPKDWEGKRDGCSCPNSD
HTAAEVLSKESQKLRNFAFERWQQREKIVRPSPPLAVCPSDSPLQFVSSGLEIEPMQSIN
LAGLAVKTSGKSILYWNNPVELTFTSVRGFGVLLLTSRLRVTVDGNSTQRMTLSVTGDTR
KLKALNIVLKGVLYQSTVAVYDEWDTIEIRFIDSVVHMHIRIRRQPLPYLYKTSSVSPPI
HERVTIVTKTFERYNKLNRFVDSVFKFYPSMTIIVADDSVHFQTVKKTNVLHYEMPPQMG
WFAGRNLAASQVKTEYLVWADDDFVFNEKTKLELFMEKLDKVELGLDLVSGHVGTNGYCG
TCILLGKNNESHCVTVMSGCNRGPVRGYPQCVQVDRPMNFFMARTKSIQQIGFDPHFKNI
GHSEFFMDGYHTLKSACCSDVSVDHIRGGSAFYMKNRKIDGQLLADYRTYIAFKYNIRCL
SIVT
>C_can|DN30744_c0_g1_i4.p1
MPISRSLTYNSIALVTALAFLIFITTFIRLGLFDVQSARHCNRRIQLDGRTLDDTCLLFN
STRSSLWGARVLQRLEDRRVAKGCSCSRALRLELQDDVLRIRKHEHNKWKGREKFIGPRP
PLAFCPSDSPLQFPSTGIEIEPKQGVTLVGLAISASQTSYWDDTVVEIVFKSMRGLGVLS
LPRQSFYSFVRMNVVVRGNNTNRMTLSAQSRHALNLNVALKDLMYQSTVAVVNELETVSV
SFLGNVFDIHIRIRRKRMPYLRKTSSVFPRIEERVTIATKTFERYDKVIRLIDSVLKYYP
SLTIIVADDSVNFRTIRKKNVLHYRMPPQIGWFAGRNLVVSQVMTEYLVWVDDDFEFDKN
TKLELFMEKLDRLDLRLDLVAGHVVSPGKTGSCGSCLQVEMDREGYCVTVNRSCSLGPLD
GYPQCVLVDRALNFFMARTKSVHEIGFDPHFNHIGHTSFFIDGYHTLRSACCSDVSIGHF
SAGSSPFYHQNRRISGQELIKHQSYILFKYNMKCISFV
```

12. NEU (1,2,3,4)

As stated by Simakov et al., cnidarian sequences indicate that NEU1 was present in the bilaterian ancestor. These sequences, and other sponge matches are distinct from the deuterostome NEU2/3/4 sequences. There is no way to date the split between NEU1 and NEU2/3/4, which could have occurred on the deuterostome or bilaterian stem. Using more sensitive hidden Markov model database searches initiated with the Pfam BNR_2 model, we identified a couple of NEU related protein sequences in protostomes (see below), but with no clear relationship to deuterostome sequences beyond general family membership. Taken together with many sponge and choanoflagellate family members, this suggests a very dynamic history for the family in the animal lineage.

Our assessment: NEU1-like sequences were present in Urbilateria, because present in deuterostomes and non-bilaterian animals. Deuterostome specificity of the NEU2/3/4 sub-family depends on the precise timing of a gene duplication event, with deuterostome specificity (if this occurred on the deuterostome stem) or protostome loss (if origin on bilaterian stem), but NEU1 is a clear protostome loss.

Protostome NEU-like sequences:

```
>La|919100968  ref|XP_013386852.1| PREDICTED: uncharacterized protein
LOC106156237 isoform X1 [Lingula anatina]
MPRLRRMESLSAYFSDGCRAQPLWFFMMVAVLPATTGKSFDGLLRRTTDGRYEAYMKPPY
KSNHASFIERLSNEDMVIAWFSGSKEGESNVAIVFARLPNGTLQWMNPRTVSQRPGYSNQ
NPVLFADNNTLFLFHTQQKASFTSTCNSDVGSEDSAQVWALVSTDGKGEKFTKPKLVLSK
AGSFDRNRIIRSLKNEWLFPTYFAGGSSKDQHSILMENAKHDPYSSWVGHAFPKSDYLVQ
PSVVRPVPGKPNLVVFFRDRRAGNIYRATSPDDGKTWTTPTKTTLPNNNSGIEANVLKSG
RIALVYNPTHHARDPLVISLSEDQGKSWKYTRTLETSSSGKNVVEYSYPSLLQDTMARIH
VSYTYNRETVKHVIIPNEQWIMKTY
>XP_034309900.1 uncharacterized protein LOC105346535 isoform X1 [Crassostrea
gigas]
MINFRDFFWFLIFAITANADPKFDGVLRNASDGTMEAYMIPPFASNHASFIEPLPNGDLV
MAWFSGTSEGESNVAIVFSQLKNNSDQWTKAQVVSQRKGYSNQNPVLFHDNKTDVLYLFH
SQQEAKKASAGIQSEDSAEIWVLSAKNVTNSTAIQFSPPRVMFKHKGSFDRNRVVVSLRN
TWLYPMYYAGGSSKDQTSNLKECVDHDVFSSWLDHPFTASNYLVQPSVVRPKKGNPRLIV
FFRDRRAQHVYRAESPDDGKTWTRPSKATLPNNNSGIEASVLSSGNLAIVYNPTTRDRNP
LSVSLSDDQGITWKYTRNLEYTKSSNGVEFSYPTLFQDSSGRIHISYTFNRQTIKHRILP
NEQWIMQK
```

Deuterostome novelties for physiological-metabolic specializations

13. PCSK9

Blast and other database search methods reveal strong hits to sponge sequences, although there are matches of comparable strength to bacteria. Phylogenetic analysis suggests all animal proteins similar to PCSK9 form a monophyletic group to the exclusion of bacteria. Simakov et al. also report this relationship with *Amphimedon* sequences, but discount it because of lack of conserved exon boundaries. While this and the presence of an additional 'CUB' domain suggest a degree of independent evolution, we consider it more conservative to hypothesize vertical descent, rather than horizontal gene transfer.

Our assessment: present in Urbilateria because present in deuterostomes and non-bilaterian animals.

Example sequence:

```
>Aq|XP_019849358.1 PREDICTED: uncharacterized protein LOC100638459 [Amphimedon
queenslandica]
MRSLQSNVKFMLLAALIYLILIKEKAEARRVIRAASGSNTTGNYIIVVTDGVNHSRFMEI
VDQVRNETLDSKIYEQVEGPFINIISARITEDAAHRLKVMDGIEFVEEETYAVKSVSWAI
DRLDQTGPTLDNTYTPEGNGEGVDVYILDTGIKYSHNDFGGRAKYPGFDPVDTADGTNQN
GLDCDGHGTHVASLAVGTNWGVAKGADVYSVRVLDCEGSAPWSVIVNGVNKAGSRIVSSN
RSGVISMSLGGGYSHTLNSALISVMNKGVPVVAAAGNERDDACNYSPASTTGVITVGGSA
KGDSLYYYTNGGTCVDVFAPGSQVTAADSSCNSCSVTYSGTSMATPIVSGSVAIHLQKNP
ALTPSQVRNKIIQDSLKNKLTYNNLKNSLRSGTANRLLHVKPKAETVTVTSTSTTTQEVT
QIITSISHATVTQQVTRTTVSIVPTTVTSVSTSHVSITSTATVAVTAIVKTSVPVTVTVT
PTPTPEEVCCSTLRNFETFEVRMMSGLSDIVNKGVETIIERSSTSSNSTCEVKAPIDGNT
TTNGTKPQAVTKSCAEIITSLPGASSGRYYLQSKDSKMNGSAAVYCELEKEIKGNRGFMR
IANVNMSDPNTDCPEGLLLRTDGNLRTCQRHQFHSGCSTTSFSSSGVEYSRVCGRIRGYQ
```

WASPNAFYDTTLYSSPKVEDDYVDGVVLTNQVNESRSHIWTFAAALDETDRNKAIVCQCS
NVNVTLNDFKTPSFVGNDYFCETGSRHQFSYGVLYTEDPLWDGKGCGKVSTCCDKGEWFC
KDVPKTSSDIELRLCGNEDRNNEDTPLDLIELYIQ

14. PADI

Blast searches initiated with human PADI1 and phylogenetic analysis revealed an ortholog in the protostome *Priapulus caudatus.* Searches against sponge sequences revealed hits to homoscleromorph sponges with comparable bit scores to bilaterians, and searches initiated with these sequences retrieved metazoan best hits. Other investigators have also recently reported PADI candidates in *Priapulus (45).*

Our assessment: present in Urbilateria, because present in deuterostomes, protostomes and non-bilaterian animals.

Example sequences:

>XP_014670183.1 PREDICTED: protein-arginine deiminase type-2-like isoform X2 [Priapulus caudatus]
MEGTSYLSERGPGASRNEGVRTINLILNDVASAVVIVKTEICVRISSVAPEGAVRYGLVS
DLDGDITVKFREESVSGYGSEEDVAPVVVAYVHVATVSRQENDKRVEFLFYNVDGEDLGK
ADLHLTCVDISLDVDADRDGIVEKNSPHKNSWKWGRDGYGAVLLVNSDCETANETEMRDT
DDELVNGPLDLLDMGRMMLRTDGPEQLPEGYSMQLYVDDKFADYVGVFYLTADTEAMMKN
KHVIGPRQRAAEVLYPGMGGEVEFAVEGLSFPDRYFEGFLHIHLALFKDDVPIYEDMVQF
RVAPWIMTPNTLEAEIVYVCDTRDNKHFVEKLRGFVEGLSGIQLVVCGLDGNRGDRWMQD
EIELGYTEVPQRQPMAVVLDSPRERGLCKFSQRQCLGPDFGYATRNTSRHKANSLDSFGN
LEVSPPVKLNGKDFPLGRILIGNALTSSLKGRRMMRVVNRFLYAQKVQPVIELFSDWLNV
GHIDEFMTFVPVPGSRQGFRLCLASPNKAYGILEKLQTDGHGDVVMFEGKRASRRSKQMK
QETVSELLANQTLRAENKKFQAYIDFNRDILKRELGLEEGDIVDIPEIFINELDPKNETI
EPQATSYFPDMVNMLVLGKNLAIPKPFGPVIDGECAFERYVRSALEPLGLSCYFLDDWYT
YHLQGGEVHCGTNTKRKPFSVKWWDLDLPRMCQ

>P_jan|DN21170_c0_g1_i1.p1
MALWDRIVRLRLETPRGSPSKSPEQEFLVTGTRLVIPFNGFYSAVPDGVVKVRISASDGL
NIKLPDSNKILEPDTLDYEQGAVMWVTSQSPSVNVGDRKVAFHFLDSSSKELAVAEIAIT
VVRLSIHADVTRDGKVNFEKSVDKRSWKWGSAASGGIGAILLVNNDRDGRRKKTDSSDSK
INGPLDLADMSRIGLIMEGPEQLPENYRLVLHTNDTTSKQIRVFEFSRGGRSSTELIGPK
RASKDVTRLWQGKGRLQLAVEGLDYPDKGFSGSGYIHVSVVEGSMTIFQDSFVFRMAPWI
MTPNTLNPRKVYVAEMVDGDNSAFIKELAALTKAAGVELEIVPSTLNRRDRWMQDEIELG
YSQAPHKSLDVVLDSPRDRGLKQYPKMKLLGEDFGYVTRGDDPANSLDSFGNLEVSPPLE
GYPLGRIIFGGAADYAPWPGRRMMKEVREFLFAQQVQSPIELFSDWLGVGHVDEFMSFVP
ADKGKYPKGFKLLLASPAACYKFLQELVRDGHGDKEMFEGKTRWNGEDATTTPKKLLQDK
RLRRENTTVQGYIDWNRDVLKRELELDESDIIDLPALFILEDDDRAGAYFPDMVNMLVLG
GHLGIPKPFGPKVGGECAFEKHVNSLLNPLGLESHYIDDWDSYHNLSGEVHCGTNTLREP
FEAKWWEMESPGQQQ*
>C_can|DN15171_c0_g1_i2.p1
MSLWDRVVRLRLNPIFATTDSAQKELLVIGNRLEVPFDAFYSAVPADVTKVSVSWSHGLD
VTLPDSNSTSEENVIDYNQGVVVHVASRTPSETIGDRKVAFRFLDSSLTELATAQIAVTV
IGLSIHADFTRDGKVNYGSDIDKLTWKWGSNENGGIGAIILVNNDADGRRKKTDVDDDKI
NGPLDFQDMSKIGVKMSGPKDLPHDYRILLHTNDDTSQKIRVFEFSRRGRTSSEVIGPAR
SSKDITRMWKGNEKVELVVEGLDYPDIGFDGSGYIHLSVIEDYMTIYQDSFVFRMAPWIM

TPNTLKPKKVFVAEMKSGDNSKFINELKQLVKAAGVPLEVVGPKLNKRDRWMQDEIELGY
SSAPHKTFSVVLDSPRDRGLKKYPKSQLLGPDFGYVTRGGGKANSLDSFGNLEVSPPVEG
YPLGRIIFGGPTDYYPERRMMKEVRDFLLAQRVQAPIQIFSDWLSVGHVDEFMSFVPADK
SKYPKGFKMLLASPAACYKLLEELIRDGHGDKKMFQGHRRWTSEDATTTANELVEDERLR
RENKTIQGHIDWNRDVLKRELKLDESDIIDLPALFTMEEGGAGAYFPDMVNLLVLDTHLG
IPKPFGPKVRGKCAFETHVNSLLNPLGLTCHYIDDWDSYHVLSGEVHCGTNTLRTPFEHK
WWEIQPAARVE

15. FTO

We identified likely orthologs of FTO in homoscleromorph sponges, with the best hit of human FTO to our sponge sequence database scoring 214 bits. There were no significant hits to bacteria.

Our assessment: present in Urbilateria, because present in deuterostomes and non-bilaterian animals.

Example sequences:

>P_jan|DN32432_c0_g1_i1.p1
MKRRRLNRDEKKSKRMKLLRELDKGIGDYIEPEDQRYQELLDCSYHKLRVIPFNRTPPHL
HSKVQTSLSHLLEAGYFTHNLVTKEAKTLITPVTRFVAGKRGLTYPYLGLRLFAFPWETD
DTVKSDAEKSLCVLGELNDYMKATGDNMWSEGDVNRFNMTLVNYMDPGHVTIPLALEPYY
HMGMCAVRWHSDQNVQDNCSIGVYSHTVGETDQSVPWSIGLKIAWDVVTSSVKIPLPDGS
CYFLLHDMNMTHQHTVIVGDKPRFSSTHRVVILEGNTLEYISERCRQAEATADRLNTEEF
LCPGDKLEVCLEIQREVEFQWIRQFWCLGMSNAKRHASFWLKHVEGMERTWENVEKAIKI
LYDRILDVTHNAKSQHKDFRSQSCDFTKIILHHLQTREKNRQDWKRRYTAPVLCYLSQSD
LPVERPAFPRDHPHFPYNQSQAIETLGSQLRDQ*
>C_can|DN8123_c0_g1_i6.p1
MIFIIKKFKININKFTGHVICLLSGIRWQQKKRRVVLSEDAKRSKKARLLRKLRDGVGQY
LEPQDEGYRELVSSAYNKFRLIPSHQTPLSLHSKVQLSLSCLLSSGYFMHNLVTKETKIL
LTPVTRFITGIRGLTYPYLGLRLFAFPWEFEGDPKSDVEKSLCVLRVLNDYMELKGNELC
GERGANEFNMTLVNYMDPDHVTIPLVTEPYYAMGTSAVHWHSDQSVRDDCSIGVYSSTVG
ESNESEPWSIGLKIAWDVTTSAVKVPLLNGSCYFLLNDMNTTHQHTVIVGDNARFSSTHR
VVVLDGNTLEYISGRCRQAEMIAKRLNGEGSVCCQDKLEECLDVQREVEFEWIRQFWSLG
RLNAERHAGFWLREIERIEQTWERVESAVKTLYDNLLERGDTKSYEDKQSQSRSVAGTMC
CAKIILQHLERRADERQQWTKRYNSSVLRYLADNDQPIKRPSFPCRHPHFSYDQTEAMDA
LKSILNKP*

16. ARSK

We identified likely orthologs of ARSK in homoscleromorph sponges, with the best hit of human ARSK to our sponge sequence database scoring 554 bits, and the best hit to bacteria scoring 371 bits.

Our assessment: present in Urbilateria, because present in deuterostomes and non-bilaterian animals.

Example sequences:

>P_jan|DN5128_c0_g1_i4.p1

MLLTIALQFSILCFLKERNALPIGNGTQQKPNIVFVICDSMDGRLVGNKDNVVKLPALDG
MTKNGVVFRNTYSNNPICCPSRSALWSGLHTHVTRSWNNHKGLPQEYPTWAVKLQEAGYT
TEIMGKTDYTSGHHTVSNRVEAWTRAVNFTLRQEGRPTPLLVGNSSTKRVSRDWRFADKA
VEWLKETGAKLEGPFLLYVGINLPHPYSTPEEGENAGGSTFKTSPYWLDFVDKSKVTIPQ
WIPMDEMHPVDFYDSKVKNCTSNFTRDEIFNVRQYYYAMCAETDAMLGQIMDGLREIGKD
KNSYVFFSSDHGEMAMEHRQFYKMTMYEASSHVPLVVTGPGVPNHQIDDVAQLIDLFPTF
MDIAGAVHPPGLNGTSLMPFLQPHVYGNKLEERPDYVLSQYHGCNVNISSFMLRTGKYKY
VAYGNGPSQIQPHLFDLSQDPDELHDLGGVKKDIVEQMDERLRSLIDYPSVAREVDAYNR
ASFQEWRDNLGEQYEHTVANLRWWMDWQKDPEGNQAYIDQWLKQSSGNLD*
>O_car|m.42407
MVLLSAVLPLGLFLCSAAVPSREKQGKNILFVICDSMDGRLIGKDVVHLPNLEKVAQQGI
VFENAYTNNPICCPSRSALWSGLHTHITQSWNNYKGLEKGYPTWAVKLADAGYETEIIGK
QDYTSGSHSVSNRVEAWTRAVNFTLRQEGRPTPKLVGNSSTIRVSADWHLVDKAKEWIMK
TASNITDKPFLLYVGINLPHPSGTPAEGENFGESTFPTSPYWLKYVQSNSVRIPKWLPLN
KFHPVDFYESAAKNCTSDFSQEEIFAIRQHYYAMCAETDAMFGAIVDALKESGHGNDTYI
FFASDHGEMAMEHRQFYKMSMYEASSHVPLVVSGPGVPAATQSKNVTQLIDIFPTFMDIA
GIEHPKGLNGSSLMAFLKPGFGSGYRDRPDYILSQYHGCNMNMSAFMLRKGQYKYVAYGN
GPEQVRPHLFDLAADPDELNDLSLQNLEVVKEMDVALRSLVDYPSVSKQVDQYNRKSFKA
WRESLGDKYDDTIANLRWWMDWQKDPEGNQKKVDSWLNS*

17. Polyketide Synthase-like, also called PKS

Searching the NCBI NR protein database with the v30045764m gene model of Simakov et al. yielded deuterostome best hits, immediately followed by a hit to *Lingula anatina* (XP_013403201.1), with no intervening bacterial sequences. This *Lingula* sequence recovers deuterostome sequences as best hits when searched against NR. (The N-terminal region of the Simakov et al. *Saccoglossus* sequence is not included in NR gene models preventing recovery of what would otherwise be one of the best deuterostome matches).

Our assessment: present in Urbilateria, because present in deuterostomes and protostomes.

>XP_013403201.1 compactin diketide synthase mokB [Lingula anatina]
XP_013403202.1 compactin diketide synthase mokB [Lingula anatina]
MPQRRKKEPCQMPTWLPENKKVAIVGIGCRFPGEINNVKDFWEVLLKGIDCTRPLPDDRF
DVNHFYHPTPKTPGKLYVRGGGYLEQDLLSFDRLFFKMPPDEANHMDPQVRLLLEVVWEA
FEDGGIPARSIRGTNCGVYMGVTASEYTTLTSARSDAISQYSNSGTNSCMVSNRISYEFD
LHGPSLSIDTACSSSLYAVHLASEAIRNGDCDMAVAGGVNVIMMPEITIGFCQAGMLAPD
GRSKSFDSSADGYCRSEGAGVIILKSLNKAVEDGDRIYAVIRGGALSNDGRTPGIANPSY
SAQVNLVYRACRHAMINPQDIQYVEAHGTGTQVGDATEANALGKTIGQIKDAKTPLYIGS
VKSNIGHCEGSSGIAGVIKVALMIHNELIPPVVHFKLGNPKIKFKELNIKVPKELIIWPY
VEEMPLIAGCSSFGFGGANAHVILEAPPSTNDLLLPEHESGPQIKVVLISAATTTALSSR
VDDFIELFRKIQDQEPERFYDVAATTCLRSHHHQHRLGVLGRDASEILHSLETFKTQKVA
AGFVSGEAVDSNTQTPLVFVFNGMGTQWWGMGRQLVHQEPTFAKTIQNFDTCLKRCGAKW
SMQWLLMNNQDEDLINQTEYAQPAICAVQIGLVEVLRKFGVRPDAVVGHSVGEVAAAYAS
GMLSFDSAVLIIYNRGKLLKKTSGSGKMLAVLHNTDAAQAKLNESKHSVYIDVAAINSPS
QVVFSGSASSVDALAEMFNQEGIRAVQLKVNNAFHSYQQEPLKEKFLQKMKPLAKQEVVN
AVQVPMISTVTNNYNHYADVNDGMYWWKNVRQQVKFQSAVDTLLDEGYSIFVEIGAHPAL
RGPIKDIIGARADKPVKVVVLETLKRPTASSPILEDLKNVVKTLTSLHCEGYPVQYEAMF
PSAYKVMHLPLYPWERITCSGITDFVRREYLFPVHSHPLLGEMQEDVSMHNNDPPFQFHW
KSKISRINTPWVSHHIIQGSVILPGTAYIETALEAVNAVRQKRETITLEDVELKRFLFPK
NGEATIHTIFQENDPTTNIKICSGDQSDYIEHVSCKVVALEEDEFKTQEVDIVREAFTLP
QGKLEHIQDSLELHYSSEEFYSNLAVGEIQLGSAFRAIQQASFNKSFTKCLIHTEAPDEI

WKEFMLYNFHPAFLDCMMQSFAVLHLLRTNSPAFPSSLKRFTYYSKKTPKRVAVYIKMLS
ATDEDQNEVLAIDADSGIVCCRIDNLSFQSLEGEHSKDPMKVWSIFWVEAALPKQNEKKG
EASPVFYLTSDNKEDLTVLQSSLKKLHRVVKILDLQDERKLNVDDIVLILISQNESHISG
PTITTKEDVWKLIKSSSSFARDVILGILNLGDVLPKLWIITVKGFQVSEGDQVSPFTAST
FAVGLSTLHEYPSARVYMVDISSLSDISTPETELMNLLCFPPFGENEFALRPKTAHSLQL
FVRRTKFQSPLTRRHPIVSHQFRLHQNKAKQIKYVYEGDTITDVQPDNSFRISIEHFIAD
GYFDDDPHFICTGHVIQSKGSEANLQDGQTVIAVFKGCLKSETLIPQENVMPLGNFETSH
AALLSLVNDAIASVIMVYSAKSVREDDRVLVCTCEESRKGLVTAQILKTLGCNVTILSYQ
CNNDCPDHEKLPCGQRISNFMEDEEVLQEDALQNIGKFNAVVATGQYLINKKGYFQCLDC
LCDFGVFVVFTQTPLKLRNFNGIRENVQFLPISAGVKQLLKTPQYLAETLKFVDALSNKL
CHLPTIGHALPASKIENKYLQECMGRVIHLDRPSMGMVSTFSEDTYKPNPEMTQCITGGA
KGMGLKIVRWLIRRGSKHVVILTRSNPTTANQSVLDQLGKNADIKLYICDVSDASAVDVV
FSEIVETQPPVENIFHCATVYLDNWMTNMSEKDWEAVMLPKAYGAVLLHQISLKYVLPLA
NFVMMSSLVALIGNAGQSNYATANSVLVSLVEMRRSMGLPATAVSFGVISEAGFAADNNL
VSVWQEKGIGHISPSQTTAVLGTMLELQYSHLGVTGHFDEAVYARKHRSFITKGAVEGDV
TLSRLQDLFSKKTLQELAVGKYSRSHQTESSIKEKLENYLSQTLGIVDISEDTSPVEMGL
DSLMATELRAFIDNEFNVSIPPVDLLNSSTTVAQLVSTICKQLHSKGDNASTEVVKGSDQ
SKEAKTSFFITEKILENPKFQLFCFPPSGGGLTSFNGWQKYFSKFHTQVYVAQLPGWEGR
RLEKPMKNMSEVIGILAREMRPLLKHGKFAFYGHSFGALLSFELAHFLNEQGVLPAHLFM
GAWIAPQIPYKETPTFRNKILDGNMDNVNFVKEAQQLTFLDGKIINNPMILRQFVPVLRA
THEMSKNYEYAPSAKLPCPLKIYGGKNDAFVAREKLTAWEDIRHKDFSFGIKMFNSGHLF
LHDIHIRALLTKDIAKTLHFV

18. NHLRC3 (NHL-containing protein ENSG00000188811 of Simakov et al.)

We identified likely orthologs of NHLRC3 in homoscleromorph sponges, with the best hit of human NHLRC3 to our sponge sequence database scoring 243 bits, and the best hit to bacteria scoring 151 bits.

Our assessment: present in Urbilateria, because present in deuterostomes and non-bilaterian animals.

Example sequences:

>C_can|DN59787_c0_g1_i1.p1
MLSMHARRVVCTAALLCLLTQFERSASRILLKYKLDATWPLEPSKFVASAQDFCTAVDDV
SQEVYVGLRGPGIPLSGPVLVFGEDGTYHRSWGHNVVDMIHGMRLQRLANGTARIWVTDL
GNGAMGHTVKMFSTDGDLLETIGTPGKAGVSLNPIQFDQVADIAIDQSDSLYIVDGDGGM
NNRLVKLDANRNLAWSVGVRGSKPSQFYIPHSVEFDPNLGMVWVADRGNNRTQAFNASDG
SFITEWTCMRPKSPCHIRLDPSGHNFIMLDLLYANIFVIPAPKDVQSVNSCHVVRQTAMY
PNNTKPHAFSISKTTWALYIGEVGANVTQKYVPYSGLSRDKHSAMFTTVTVFEPFLLCLL
FVALLLWVVRRRLRSGGKPHNQRVGLIGETIWHK*
>P_jan|DN7480_c1_g1_i3.p1
RMAERARAVLLVSFLSVSWSFHLPRNPLSAFEYKLDATWPSATTQFVRSAQDFCTAVDDI
RHEVYVGLRGPGIPESGPVLVFGEDGTYRRSWGHRLVDMIHGIRLQRLVNGSLRVWVTDL
GNGPLGHTVKMFSSDGVLLETIGTAGEPGTSLKPIQFDQVADVAIGQNNSLYIVDGDGGM
NNRLIKLDANRKVMWSVGSRGMKPSQFNIPHSVELDPRLDVVWVADRMNNRTQAFNTTDG
RFLAEWTCMRPLSPYHIRLDPSGHYYIMLDLASARLLVLTAPDDIKALNICRVVSETVMH
PNNTKPHAFSVSRSTGALFIGEVGANVTQKYIPCNTSNRHQAHHPDRHEMSFVCTTVFEP
FMLFLVFAVVLTWILQRRMRSGGRAHYHRVQMKDGSSWHK*

19. Choline monoxygenase-like (also called CMO)

Sequence searches initiated with the *Saccoglossus* protein (XP_002738379.1, see below) retrieved strong matches to deuterostomes and the protostome *Phoronis australis* (349 bits). The best hit to bacteria scores 351 bits, but the *Phoronis* sequence searched against the entire NR database of the NCBI retrieves animal sequences as best matches.

Our assessment: present in Urbliateria, because present in protostomes and deuterostomes.

Example sequences:

```
>Sk|291236901  ref|XP_002738379.1| PREDICTED: choline monooxygenase, chloroplastic-like [Saccoglossus kowalevskii]
MPSRRLAVLKRATSSSSNLRTGDTNDPSFDLRREVAKFDANIPVEAATTPPSTWFTRPEF
HQLEVHTVFHDAWMFAGRVDQITKPGKFFTGTIGKEPYIVLRDENNELKAFYNVCRHHAM
QIVNDTEGTVKDLQCPYHGWTYALSGRLKKATRLRGIKNFSARNFGLIPMMVKAWGPLIF
IRPNKISEEKDNFFEDLESLKNRLDGIGFSSGMKFMKRITYTLNCNWKVFVDNYLDGGYH
VSTAHKDLSVALDASSYRTAVHEWHSIQSVSAVGSEERVCGDAIYAHIFPNLMINRYGPW
IDTNVALPLTHNTCMIVFDYFLEEDYIQQKSEEDLQNILDTSFTASDKVQQEDIFLCEGV
QRGLESSAYDLGRYAPGVEFADHMFHVKLAELFRRHLKIP

>Pa|g2550.t1
MNRLLSAGLLRSLTTRFSVFQAENDVQRIRWIPFSTSMNGMNTAQRHEWIATVKGEVNRF
DPSVPIESAITPPASWYINPSFLELEMETVFRNNWIAVGHVGQVARKGQFITGTIGKEPY
IVTRDQNDDLQAFFNVCRHHASVILEEECGEASQFVCRYHGWTYALDGRLAKAKRLKNIR
DFKPRDYGLKKIAVQTFGPVIFLKLNEKVDVNLSSELEELKASLDEAGSENSLRWVKRAE
FDLDCNWKVLVDNFLDGGYHIEECHPKLDSILVSNSYKTTCSGLFSIQSSMGTGERHGNN
AIYAHIYPNCMINRYGDWMDTNYIFPVGQEKCRVVYDWFLSERVFSELSEEEVEEKIKSD
LENSCLVQIEDNEICARVQKGLYSRSYQYGRYAPLVEMAQHEFHRKLYSDYGLELD
```

20. Ectoine synthase

Sequence searches initiated with the *Saccoglossus* protein NP_001171779.1, L-ectoine synthase-like retrieved strong matches to anthozoan proteins with a best score of 142 bits, while the best hit in bacteria scored 124 bits. Searching the NR NCBI database with the *Exaiptasia* hit (below) showed best matches to *Saccoglossus* and other deuterostome proteins.

Our assessment: present in Urbilateria, because present in deuterostomes and non-bilaterian animals.

Example sequences:

```
>Sk|296434217  ref|NP_001171779.1| L-ectoine synthase-like [Saccoglossus kowalevskii]
MIVCHSEDVAVATLPNIEMKGKLLTDVEDGMGFSAYQVVAEEGETLKLRSSDSGDLDHIY
YCISGTGDVKASNGCHYKLKPDCVVAFSSSVSAELIVETRIRLYVLYCDDINPSSERSVV
KSLDEIVGTERDVDFKRGHSRRFLLKVDGFAITITSTAVMFTGDDPTKLEYRNHAESAYY
ISGKVSYSWNEGANKIEARITPDDGTVYNMNAHDKHMVSVHEDCIALCVFYPALRGNENH
TYDGGYSCYDA

>Ep|XP_020909527.1  uncharacterized protein LOC110247442 [Exaiptasia pallida]
```

```
MKSRILSELPDEQLGQTTLSGKILTFLEDNVGFFEAQFLLEKDQSHDLTPVYQDHNHIYY
CINGKASVKIDQGDNQFTLTDNTLLALSPSTKASITALASTRLVVVSVPGLPNQQPXPYF
LSLEKISGTDRDVNFGAGQSRRFLKQSDGFNISVHNTVPXLKTKVNLEYKNHFEANLLCK
GRVQYWWNNETETAVFDEKDSVTGDGIMILLDKHDNHHNEVLQEDSHSICVFYPPLKGDE
KHNFSGSGSSY
>Pd|XP_027037521.1  uncharacterized protein LOC113665976 [Pocillopora
damicornis]
MKVRVADDLPIVPLGSSEIQGKILVSLEDGVGCAMYEFYAQAGDEVTLIAFHRNHNHIYY
CVEGRCVIHDGATKQLPFTADHLLALTTDKNVHIRIQEPMRIVAVSVPHDTSVSPPPLLM
KSLDEVTDSDRNVPFEDGLSRRFLAAPDGYNISVHNTFCSSSFSKHLQYLNNKELVYVIK
GQGEYVWENGQCRHDFDSEKHHGTMFLVGNNAHKMTIGARDTIAICLFYPPLMGNERLKT
GKEGGSSY
```

21. Ectoine Hydroxylase

Searching the NCBI NR database with *Saccoglossus* ectoine hydroxylase yields hits to 3 Saccoglossus sequences and 1 from *Branchiostoma*. Phylogenetic analysis of top 500 hits suggests these animal sequences form a single clade.

Our assessment: deuterostome specific

Example sequences:

```
>APU50791.1 ectoine hydroxylase-like protein 165 [Saccoglossus kowalevskii]
MYSRIQRIARFPRLVPAIKLSHRVTSQVNTGRLYCSATKDLYPTRENECKVLPRLDPVVW
NNPDGKFDGPLSSAELQEYEKNGYLILKDLFSPEEMEPVIRECKAVQKSIECNQYDIKTG
EEAKIAMELNSTKLRSYVYAHEDIESVKKLSRNAKLVNRARQILADEVYILQSRVNYHQA
YVGQGISWHHDFDVFHADDGIPRMRALTFAVMLDKNTPETGATMLVPGSHRNFLTSLGPT
PDNSWVLSITSKSYAGIIEQHMLTPVIESHGIEHATGDVGTVYLFDINTIHCNNVNVSPF
NRVNVFLLYNSIKNKAIKPFSAPHPRRPEFIATKDNAVPIVPE
```

22. Histidine methyltransferase (HMT), bacterial-like, also called methyltransferase

Searches initiated with the Pfam Methyltransf_33 HMM, scoring above the gathering threshold cutoffs ('--cut_ga'), retrieved hits to deuterostome species and sequences from the protostome *Crassostrea gigas*. Simakov et al. also report the presence of *C. gigas* hits, and their argument for this gene being deuterostome specific rests on the oyster sequences being contamination. Using current sequence databases, similar sequences are also present in other bivalve molluscs.

Our assessment: present in Urbilateria, because present in protostomes and deuterostomes.

Example sequences:

```
>Sk|585695626  ref|XP_006822057.1| PREDICTED: uncharacterized protein
LOC102808773 [Saccoglossus kowalevskii]
MEKKIKEGLLSTPKYVPHWYLYDTQGSKLFEKNAMENPLYHIYRTEKTMLEQYSDLIISD
LGDDIVLCELGAGSLTKTTHIISALLNKNKDLTYIPIDIAGDFMLQNATVLQEQFDSVLV
EPFVGDYNDGLTYLRSIRKGKLLIFLGNSLSNIPLNKMENFLSRVLETMGEKDRFLIGID
LTQDKDKILAMHSDPKMGISFNENLLNRLNREFNANFKKENFQLHCNYVIRDDMCGTVKH
AQYIQKALKSTCEHVVHLDTLGLTIHFKEGELLYRHELVNGSLKWSWNQFEDTMKRNGLY
```

LEKTWSDDANSYGLALLKRM

>XP_034301350.1 N-methyltransferase dtpB isoform X2 [Crassostrea gigas]
MDVKELLVKGLCSTPKYIDMSCRYDKQGSIYDDQCQDIEDFYHYKAEEAIMRCNAKDAAL
QLKTPLRLFDLGCGSTTKSQYLINELLANKVNVEYTPIDVSKDFLQHACETLKNIYGNRL
KVDPIAGDFMNVIPQIGKYRGRKVLLWLSGLQCFPLDTQHQLLSKISESMEGDDSLLMTA
DITQDKAVIEKAYLDFDDSKPFAKLYTNGVHVANRELGGNIDLSQFILEGRHVEDKDVSR
ASYNQVWLRSLCNQSYYLEEVGKNVTFEEGEKLHLYSGNGISHKYTIPQLENALASVKLR
VLKKWDNGNSVLLLSTRENEL

23. Aromatic amino acid decarboxylase family, microbial-like; AAADC

Searches initiated with *Saccoglossus* tyrosine decarboxylase (XP_002731852.2) revealed hits to other metazoan and bacterial proteins. A tree reconstructed from these hits focussing on the Pyridoxoc_deC Pfam region revealed a small clade derived from *Saccoglossus* and *Branchiostoma*, nested within a bacterial clade, to the exclusion of other metazoan genes.

Our assessment: deuterostome specific, because shared by *Saccoglossus* and *Branchiostoma* nested within bacterial clade.

>XP_002731852.2 PREDICTED: probable tyrosine decarboxylase 2-like [Saccoglossus kowalevskii]
MSLVTEDLLQLFEENIERCGLVNSHGGHMAYVNSGGVFPSALGDYLAAVVSPYSAVFHAS
PGSVCLENMVINWIGRTFGFPEGFAGNLTSGGSASTILALATARDSKNVKAVDFSRCVVY
MTQIAHYCVEKALNTIGMREAVRRTVPTDTSTYKMNAEELKIMIEKDKNNGLIPFLVYAT
AGTTDLGSIDPLNEIASIARQHSLWMHVDACYGGFFIMSNEAKPRFRGIERCDSLAVDPH
KSLFIPYGCGCVIVRQGSKLKYSNALNRPAFYLEDFFKDEREDEPSPCHLSFELSRHFRG
VRVWMPLKLFGLAPFRDALTEKILLARYFHEQLAERGDFQLGPYPELSVVVFRYAKAPND
TEKFNEQLLDSLMKDGRVALASTRLRGVYHLRVCILCFRTHLKEVDFLLEMVDKNVETYL
KSVSKKPWESQQLS

24. 5-methyltetrahydropteroyltriglutamate--homocysteine methyltransferase-like

Blast searches initiated with the *Saccoglossus* sequence (XP_006818155.1) reveal likely orthologs in the protostome *Lingula anatina* and several cnidarian species. With current sequence databases, the gene no longer appears to satisfy criteria for deuterostome specificity.

Our assessment: present in Urbilateria, because present in deuterostomes, protostomes and non-bilaterian animals.

Example sequences:

>Sk|585672030 ref|XP_006818155.1| PREDICTED: 5-methyltetrahydropteroyltriglutamate--homocysteine methyltransferase-like [Saccoglossus kowalevskii]
MPLTTTVIGSYPKPDYLGIPDWFDGLTSTERYSAIWNRRPHDRRIKGFNFNMLTETSTRN
GAWTAKLPTITGKIELMDGNTWAAEEWRGAQNLTDTPVKFTIPGPATLIGSTHNAFYDDK
RTLSEDLVKVINYQIRALAKAGCRHIQLDEPVFARFPDTALNYGIEHAERCFDGIGLEVE
KTVHICCGYPCYLDQQDYQKADQEAYFKIIDKLDSAGFDAISLEDAHRYNDLRLLDRIKK
SKVVLGAVTIANSRVETVEEIRNRLEEVLRHIPASRLMVSPDCGLGFLPPPILKEKLANM

```
VAAAKSIQCDPEVIC

>La|919087672  ref|XP_013380915.1| PREDICTED: 5-
methyltetrahydropteroyltriglutamate--homocysteine methyltransferase-like
isoform X1 [Lingula anatina]
MAGTQGILTTVIGSYPKPDYLKIPDWFRSSHTGYSSDQYDEYVRRKVRAEESVVEKALAE
CMEEQTALGIDVITDGEIRRENYIYPFCRHLKNFSFEELTFKTYRNGACSGQVPTVIGPL
EPTSADPWCATEWQESQAMTKTPVKYTMPGPMTIIGSTANKFYQHDKELSEVLVKLVNRE
LLALAAAGCKYIQIDEPVMVRNPEIAMDYGIDHLARCFEGVGPEIEKIVHLCCGYPNCLD
QTDYLKADPSVYMDLAEKLDNAQFDAVSIEDAHCHNNLALLEKFKKTKVIFGVVAIAKSQ
VETVEEIKSRVMEALKHIPKERIILAPDCGLAFLPRNILRQKLSNMVTAAKSIV
```

25. Major Facilitator Transporter algal-like, MFS algal-like

When searching with the major facilitator transporter algal-like from *Saccoglossus kowalevskii* (NCBI accession: ALR88600.1 see below), we were not able to detect non-hemichordate hits within the animals, and indeed only hits to hemichordate sequences are recorded by Simakov et al. As such, on their terms, this sequence does not qualify for the general classification of a 'deuterostome specific', but rather is hemichordate specific, and is uninformative for deuterostome phylogeny.

Our assessment: hemichordate specific, not deuterostome specific.

Example sequence:

```
>ALR88600.1 major facilitator transporter algal-like [Saccoglossus
kowalevskii]
MRSNKLSREDIPGDEIDNDQSDEKVPLLSDNVTETTKEKITRVYGALKPFVIISSSYLLY
TITDGAVRMIVLLHAFSLNFTALETAIMFSLYELAGVFTNLVAGVAGARWGIKSTLITGL
CLQFVGIGMLFGWQENWTKSIGIVYVTVAQMFCGISKDLVKLGGKTVTKLVTPEDKQTRL
FKIVSLLTGWKNTLKGVGYFLGSALLMINYYLSLGVMCVLIVCALPWAICGLSKDLGRTR
RENIKLSTIFKKNFNVNILSLARFFLFGSRDLWFEVPLPFFLRSDDGIGWERAAVGAYLA
GWIIIYGQVQSWSPQLLLKPLRQSPANKWVCALWGFILIVCPVFLGCFLQFSHAIQEHVV
EIMTAAITVGLIMFAVVFAVNSAVHSYLIVKYSEGDKVAMNVGFYYMANAMGRLTGTLLS
GVLYDYVGAATSTAQGFASCFWVSVGFAVVAACVALFLNDNSGGLQCGPCLNCLNSRSGT
GEGRYEVIDNKDDNEPTQSSAVTHTA
```

26. Multicopper oxidase (MCO); also called Bilirubin oxidase-like

We find similar sequences to *Saccoglossus* multicopper oxidase in tunicates, amphioxus, but not protostomes. We also find proteins with the same domain structure in *Monosiga brevicollis* and other choanoflagellates. As choanoflagellates are the sister group of animals, this raises the possibility of presence in non-bilaterian animals, but we are unable to produce evidence that the choanoflagellate sequences form a monophyletic clade with the animal sequences.

Our assessment: of uncertain status.

Example sequences:

```
>ALR88601.1 multicopper oxidase-like [Saccoglossus kowalevskii]
```

MKTWHIFLALFVNALAIHDFQDLRQPTEIISQNGALNFTFTVDVFRYIGQVNYTTRAYYY
HGVGSIPGPTWRVKAGDTITFTLKNALPPGQSTEYEHNTMHSPDTTNMHTHGLHVNPYED
DVFIEVHPEEQHVYTYVLPDNHAPGMHWYHAHHHGATAFQVIGGLAGAIIVDPVDDSIIP
PELSNMDEIVMILQHMKFDSDPGNGCPNSENFVNAFRPYSYLEISDEIGDTLDVNPSLAN
TSLADFYVVNGQYQPRIQMRPGEKKVFRLLHAAGTHTLEIEIPGCNIYRISRDGVYRSEP
TDAVDVIVLIQASRADIIVDCPRPGTFILKSTIDPSRDNIVSSNVLRYFQDPILTIEVSG
DELIMPLPSQLPPLPSYLSDLQNVSRQDIAGQYRVDFGQLRGPETCNFGINGMLFRGPDV
YDHIMTLDTIEEWMIKDTGPESHPFHLHVNHFQVISTTYQDDPNQIVFEIGEWRDTLAAR
DVTTIRFRTDTFPGVLVLHCHYLRHEDLGMMQVTYIESSPETDTGDHGTGSIGQEAEPNR
GGIKHSESIELHESDINSSGQFKSSTYVFFTTVMIVVLSII

>XP_001746360.1 uncharacterized protein MONBRDRAFT_26023 [Monosiga brevicollis MX1] >EDQ88747.1 predicted protein [Monosiga brevicollis MX1]
MRQTSLLGLVALLLCGLATGLHSAEDTSTDAAATADRARAMQMLPAKRFSASHPAALPEV
LNSHLRRHAARYGHAVRACEAWSTSDLNAVLAELVQQGLADDGARSLLALYAQRGEPRRL
RLSSLAAYSAAWQQEEELIDRLGAASREAQLLRAGKCYEASMLYIHGASSKVKQGLSTAF
NVPLLPEGPLATLVTDHISAMRLTPTADPATSSPIDSNVLNTVQDQVVCVRCHVTPNATF
SPRQYDVVVNGSHPLLRSPQVLRSAQGVLNATLDVGVARLSGGPLEFNVRAYNGGTPGPT
LWVQPGDSIHVTLNNHLEAPDACQAGPSGAGPDSNFYRCPNTTNIHLHGLWVTPHDVFRG
IGPGGRLTQTYSLTTDHAPGTNWYHPHFHGSVSLQLAMGMAGAVVVDDPPNSLPPALEAL
RSRDHVMLLQSIEYFNYTQDDINCPYEDGLSHCHNMDGAGSLATLRAFSSDALPLNMERR
DPGYGYPIDGVYFLLNGQFQPVVTMQPGEYQRWRLVNGAHQASLSLSVPGCTVWLLSMDG
VYLSSPRLKNTSYPLVLAPGARGDVAVSCAMPGLFELRSNENPDVWNLGAQEGFNTGVIA
LVNVSGPNLNMSAPTRLPSRPAYLQDLRPASVPAGNTRNVSWDLIRLINNTAGLTDVPIY
GVSQQAFNASNPSRDCINARSGVQEWQLVNPIWICNASVTTCLSTMDRNAASGALLRDYQ
EPVTLSHGFHLHTFKFQVASDSNGGVSADYELGDWRDTVTTPLAGWVRIRWQPQTFEGVI
PWHCHMSVHSDRGMIGASTVASQCPAAPNAVSRARAAAQARARPAMAHLASGNWEPRLGN
FTWNVSTHSPAAQALFNEATMMAFGFARDAASATYLRALQADPTCAMCAWGVAYANGPFL
NHPECSNATCGLGFEYAELAATLAANHSVTAAEAMLIRGMQQRYSQDPKANQTAHFMAYA
QALNATALVHSDPTLAAFAAEAYMLLHCQDDGYHFNFPNGTPVPDLAWVIDLLGSYLFYP
NTNDVLPGPRHPFVSHLYIHAVEPSGAGMGPNAAGRAFGVAQRLSSVLPNVTSLWQHLQH
MPAHIFLRTGYYGLGVAANMIAHASDATWLNHSLVPYGPGHNLIFLIYCACMDGQSSTAI
HYGEVVRQVYQAAPDRPDGPGADLAYNWPATTFVRFGSWDAILAADWFTMPRPFPYQQAL
AHYAIGLAQAHTGNVTGARASLQNLQRVQATLTGRAATYCRVANWTLGAAVARATGPAGV
PTALSLLSTAVAEQMGWPYDEPPDFHQPIQQCLGQLQLELGNYSAAYDTFQANLAHYPNN
GWALWGLLQAVQHLPYDPSRPSAASITTRLSAAWERADVPLTSACALIHFP

Deuterostome novelties without eukaryotic or prokaryotic homologs

27. FAM198A

We only detected deuterostome hits for searches initiated with human FAM198A.

Our assessment: deuterostome specific.

28. C9orf9 (Rsb66)

We only detected deuterostome hits for searches initiated with human C9orf9.

Our assessment: deuterostome specific.

29. MREG

We only detected deuterostome hits for searches initiated with human MREG.

Our assessment: deuterostome specific.

30. SMIM19

We only detected deuterostome hits for searches initiated with human SMIM19.

Our assessment: deuterostome specific.

31. EFCC1

Database searches initiated with human EFCC1 detected a likely ortholog in the protostome *Priapulus caudatus*, with no significant hits to non-animals.

Our assessment: present in Urbilateria, because present in protostomes and deuterostomes.

```
>XP_014670810.1 PREDICTED: EF-hand and coiled-coil domain-containing protein
1-like [Priapulus caudatus]
MEARSVSRRTEWVVSALAHHYGFDQGAPNEIVVLSLGVDQYIQEIFNNLDCAGAGVVSAD
QFRALCDVLAVGSPPSPDDLPTSLDFKQFHARLCEHVLATFGVRDFRPSSARVADDDEEY
VTTSIKLNQATSLRGGGGGERPLAATACITCSMCVRELVNSLLLNKCKMSFAGAMRRGGG
GAALTAEQGDCAAKIELIWRAIVDSYETLQQRVVAQEEEVEGLRELVEDLRAALQSGDAR
CLAFQVELRRTRQLQRQRQRDPCRRCVCDGGAPAPTIHRSVKTIEIRNGRVHSPDDDDHH
QVAPPLRGDDQQAAPPTLAERYRHYSPRCMERLVREMARVRGNRDVQLQEALYVTEDLQY
EARKRRRAIERLQREADELVALQSRTRDGLREARVYVGDGLERVLELESEARELPLLREK
VRQLTKKATSPQERNQCSSSNGRSSPMGQCCSPKCPSPDILDHRAVEGQSGSEDDISTGR
DDVTIPDDVITPADITKIQAEVTSLKRQLADCHFKAEEVNASMREELANKQFECDEVLVE
LQQSENERARLTIIEHGLRDALHLLNGLKSNLVSRRTLGKMVMDALDRSETMTSADEAVQ
VFVTSLQTIAAGSALLHSSASEDSSPNTTPKRRIPVIREDIISS
```

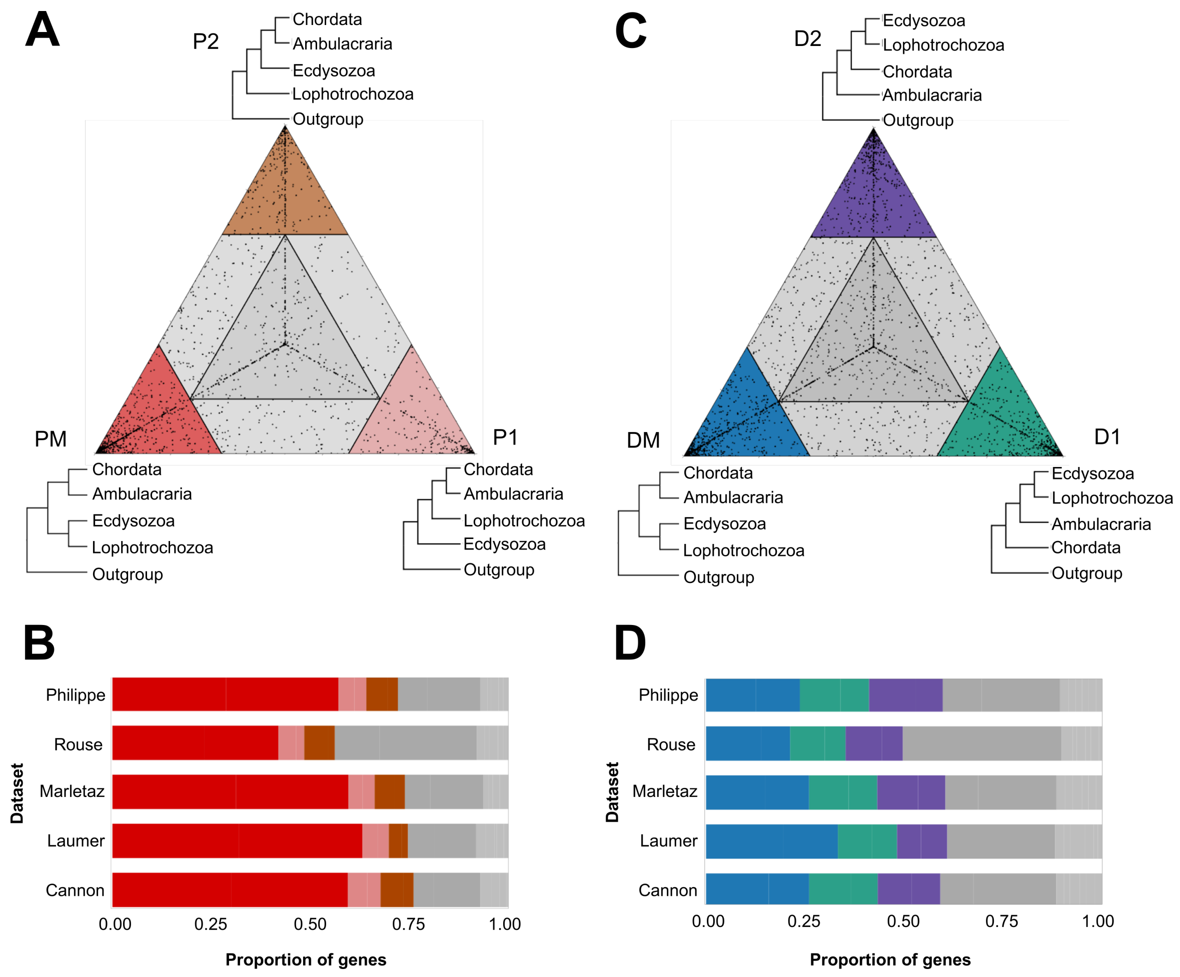

Fig.S1 Support of individual genes in each dataset for the monophyletic Protostomia and Deuterostomia and alternatives when *Xenoturbella* is excluded. Triangular plots showing relative support for the three alternative topologies shown at the corners of the triangles. Genes in coloured corners show a high preference for the corresponding topology. The numbers of genes found in the different coloured sectors of the large triangle are shown below. A. Triangle plot comparing support for monophyletic Protostomia (PM) versus two alternative topologies with paraphyletic Protostomia (P1, P2). B. Bar plot showing that across five datasets the majority of genes strongly prefer the monophyletic Protostomia topology. C. Triangle plot comparing support for monophyletic Deuterostomia (DM) versus two alternative topologies with paraphyletic Deuterostomia (D1, D2). D. Bar plot showing that across five datasets a minority of genes strongly prefer the monophyletic Deuterostomia topology over paraphyletic topologies or the grey areas.

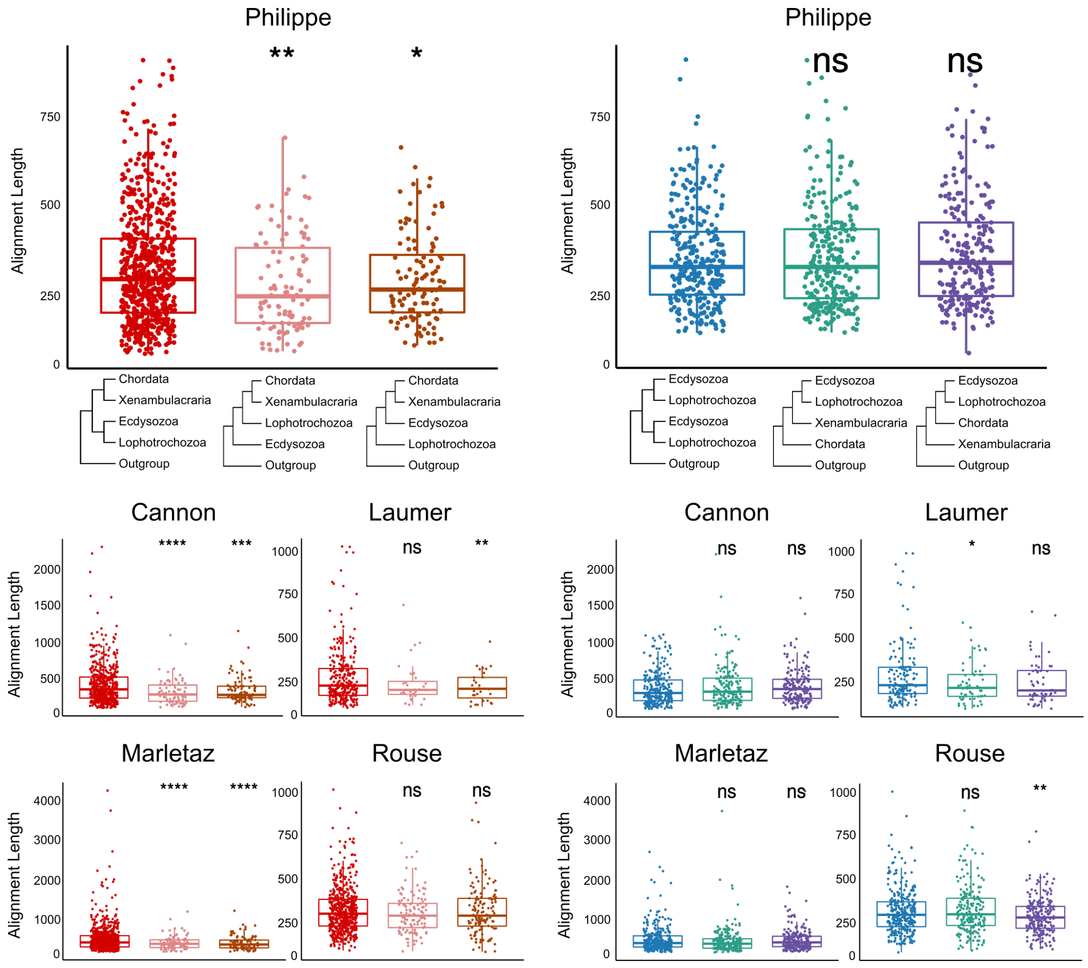

Fig.S2 Longer genes tend to support monophyletic Protostomia but not monophyletic Deuterostomia. Box plots showing the distribution of alignment lengths for genes strongly supporting the three alternative topologies shown (sets of genes selected using the data shown in Fig. 2). Longer alignments are expected to contain more phylogenetic signal. Asterisks indicate significance at $p < 0.05$ using a Welch t-test for equal means. Left: Data from all five named datasets comparing the lengths of gene alignments supporting monophyletic Protostomia versus those supporting two alternative topologies with paraphyletic Protostomia. For most datasets the genes supporting monophyletic Protostomia are significantly longer than the genes supporting the alternative topologies. Right: Data from all five datasets comparing lengths of gene alignments supporting monophyletic Deuterostomia versus two alternative topologies with paraphyletic Deuterostomia. For most datasets the genes supporting monophyletic Deuterostomia are not significantly longer than the genes supporting the alternative topologies.

Dataset	Topology	Proportions	Alignment Length	Monophyly Score
Cannon	PM	61.75%	426.2	0.634
Cannon	P2	11.01%	346.8	0.594
Cannon	P1	9.08%	324	0.584
Cannon	Px	9.99%	245.5	0.577
Laumer	PM	65.88%	269.6	0.628
Laumer	P2	7.35%	214	0.589
Laumer	P1	7.58%	232.4	0.55
Laumer	Px	12.56%	195	0.568
Marletaz	PM	61.50%	408.7	0.564
Marletaz	P2	9.20%	309.3	0.554
Marletaz	P1	7.92%	310.8	0.559
Marletaz	Px	14.22%	215.3	0.536
Philippe	PM	58.65%	323.4	0.626
Philippe	P2	9.55%	293.3	0.59
Philippe	P1	8.70%	277.7	0.582
Philippe	Px	14.32%	224.9	0.564
Rouse	PM	46.94%	340.3	0.494
Rouse	P2	11.46%	339.8	0.474
Rouse	P1	10.02%	323.8	0.466
Rouse	Px	23.01%	321.1	0.478
**Average**	**PM**	**58.94%**	**353.64**	**0.5892**
**Average**	**P2**	**9.71%**	**300.64**	**0.5602**
**Average**	**P1**	**8.66%**	**293.74**	**0.5482**
**Average**	**Px**	**14.82%**	**240.36**	**0.5446**

Table S1.Proportions, average alignment length and monophyly scores of genes supporting the monophyly of Protostomia and its alternatives. PM indicates the cluster of genes that support the monophyly of Protostomia. P1 indicates the genes that support the protostome paraphyly where Lophotrochozoa are sister to Deuterostomia, and P2 indicates the genes that support the protostome paraphyly where Ecdysozoa are sister to Deuterostomia. Px indicates the cluster of genes that show no preference to any of the three topologies.

Dataset	Topology	Proportions	Alignment Length	Monophyly Score
Cannon	DM	28.60%	389	0.621
Cannon	D2	22.02%	424.7	0.638
Cannon	D1	20.09%	419.5	0.622
Cannon	Dx	17.25%	264.3	0.58
Laumer	DM	35.07%	286.2	0.603
Laumer	D2	14.93%	265.9	0.6
Laumer	D1	15.17%	244.7	0.599
Laumer	Dx	22.99%	189.8	0.617
Marletaz	DM	27.34%	412.3	0.562
Marletaz	D2	18.99%	407.3	0.58
Marletaz	D1	20.27%	362.6	0.558
Marletaz	Dx	20.70%	240.7	0.533
Philippe	DM	25.58%	309.1	0.617
Philippe	D2	20.55%	324.8	0.615
Philippe	D1	23.02%	314.3	0.612
Philippe	Dx	21.06%	237	0.577
Rouse	DM	26.66%	350.4	0.48
Rouse	D2	19.35%	323.6	0.496
Rouse	D1	17.32%	362.7	0.496
Rouse	Dx	24.28%	297.1	0.484
**Average**	**DM**	**28.65%**	**349.40**	**0.5766**
**Average**	**D2**	**19.17%**	**349.26**	**0.5858**
**Average**	**D1**	**19.17%**	**340.76**	**0.5774**
**Average**	**Dx**	**21.26%**	**245.78**	**0.5582**

Table S2.Proportions, average alignment length and monophyly scores of genes supporting the monophyly of Deuterostomia and its alternatives. DM indicates the cluster of genes that support the monophyly of Deuterostomia. D1 indicates the genes that support the deuterostome paraphyly where the Xenambulacraria are sister to Protostomia. D2 indicates the genes that support the deuterostome paraphyly where Chordata are sister to Deuterostomia. Dx indicates the cluster of genes that show no preference to any of the three topologies.

Dataset	Genes Supporting topology 1	Genes Supporting topology 2	P-value
Laumer	**DM**(148)	**D1**(64)	0.04945*
Philippe	**DM**(300)	**D1**(270)	0.6855
Cannon	**DM**(252)	**D1**(177)	0.241
Marletaz	**DM**(321)	**D1**(238)	0.08241
Rouse	**DM**(314)	**D1**(204)	0.3317
Laumer	**DM**(148)	**D2**(63)	0.4596
Philippe	**DM**(300)	**D2**(241)	0.2372
Cannon	**DM**(252)	**D2**(194)	0.1436
Marletaz	**DM**(321)	**D2**(223)	0.8731
Rouse	**DM**(314)	**D2**(228)	0.008017*
Laumer	**PM**(278)	**P1**(32)	0.1285
Philippe	**PM**(688)	**P1**(102)	0.001891*
Cannon	**PM**(544)	**P1**(80)	0.00001572*
Marletaz	**PM**(722)	**P1**(93)	0.00001684*
Rouse	**PM**(553)	**P1**(118)	0.1573
Laumer	**PM**(278)	**P2**(31)	0.004006*
Philippe	**PM**(688)	**P2**(112)	0.02411*
Cannon	**PM**(544)	**P2**(97)	0.0002559*
Marletaz	**PM**(722)	**P2**(108)	0.000005674*
Rouse	**PM**(553)	**P2**(135)	0.9662

*Statistically significantly different

Table S3. Results of t-test comparing alignment length of genes supporting the monophyly of either Protostomia or Deuterostomia and the two alternative topologies.

PM stands for Protostomia monophyly, DM for Deuterostomia monophyly, P1 and P2 the two paraphyletic alternative topologies for Protostomia, and D1 and D2 the two paraphyletic alternative topologies for Deuterostomia.

		True Tree: D1			True Tree: D2			True Tree: DM	
**Dataset**	**Model**	**D1**	**DM**	**D2**	**D2**	**DM**	**D1**	**DM**	**other**
36-taxa-50K	LG+F+G	60	40	-	88	12	-	100	-
36-taxa-50K	C60+LG+F+G	100	-	-	100	-	-	100	-
35-taxa-50K-noXeno	LG+F+G	92	8	-	90	10	-	100	-
35-taxa-50K-noXeno	C60+LG+F+G	100	-	-	100	-	-	100	-
23-taxa-50K-no-longs	LG+F+G	100	-	-	100	-	-	100	-
23-taxa-50K-no-shorts	LG+F+G	-	100	-	-	100	-	100	-
36-taxa-10K	LG+F+G	70	30	-	96	4	-	100	-
36-taxa-10K	C60+LG+F+G	86	14	-	100	-	-	100	-
23-taxa-10K-no-longs	LG+F+G	93	5	2	100	-	-	100	-
23-taxa-10K-no-shorts	LG+F+G	-	100	-	-	100	-	100	-

Table S4. Percentage of simulation replicates supporting alternative topologies for the Deuterostomia clades under three different true tree hypotheses for different datasets and models. The datasets are based on a reduced version of the Laumer dataset consisting of 36 taxa covering all major branches of the phylogeny. In the first six rows, the results are based on a subselection of 50,000 sites and on the final four rows the results are based on a subselection of 10,000 sites. For both the 10,000 and 50,000 site samples we repeated the experiments after removing the 13 longest protostome and outgroup branches ("no-longs") and the 13 shortest protostome and outgroup branches ("no-shorts"). For the 50,000 site dataset we repeated the analyses after removing *Xenoturbella* ("noXeno"). The two models used for the inference of the trees are the LG+F+G and the C60+LG+F+G. The three tree hypotheses assumed for the simulations differ only with respect to the relationships of the deuterostome clades (DM: monophyletic deuterostomes, D1: Xenambulacraria are sister to Protostomia, D2: Chordata are sister to Deuterostomia).

REFERENCES AND NOTES

1. P. Kapli, Z. Yang, M. J. Telford, Phylogenetic tree building in the genomic age. *Nat. Rev. Genet.* **21**, 428–444 (2020).

2. J. T. Cannon, B. C. Vellutini, J. Smith, F. Ronquist, U. Jondelius, A. Hejnol, Xenacoelomorpha is the sister group to Nephrozoa. *Nature* **530**, 89–93 (2016).

3. C. E. Laumer, R. Fernández, S. Lemer, D. Combosch, K. M. Kocot, A. Riesgo, S. C. S. Andrade, W. Sterrer, M. V. Sørensen, G. Giribet, Revisiting metazoan phylogeny with genomic sampling of all phyla. *Proc. R. Soc. B Biol. Sci.* **286**, 20190831 (2019).

4. H. Philippe, A. J. Poustka, M. Chiodin, K. J. Hoff, C. Dessimoz, B. Tomiczek, P. H. Schiffer, S. Müller, D. Domman, M. Horn, H. Kuhl, B. Timmermann, N. Satoh, T. Hikosaka-Katayama, H. Nakano, M. L. Rowe, M. R. Elphick, M. Thomas-Chollier, T. Hankeln, F. Mertes, A. Wallberg, J. P. Rast, R. R. Copley, P. Martinez, M. J. Telford, Mitigating anticipated effects of systematic errors supports sister-group relationship between Xenacoelomorpha and Ambulacraria. *Curr. Biol.* **29**, 1818–1826.e6 (2019).

5. F. Marlétaz, K. T. C. A. Peijnenburg, T. Goto, N. Satoh, D. S. Rokhsar, A new spiralian phylogeny places the enigmatic arrow worms among Gnathiferans. *Curr. Biol.* **29**, 312–318.e3 (2019).

6. G. W. Rouse, N. G. Wilson, J. I. Carvajal, R. C. Vrijenhoek, New deep-sea species of *Xenoturbella* and the position of Xenacoelomorpha. *Nature* **530**, 94–97 (2016).

7. J. Felsenstein, Cases in which parsimony or compatibility methods will be positively misleading. *Syst. Biol.* **27**, 401–410 (1978).

8. N. Lartillot, H. Philippe, A Bayesian mixture model for across-site heterogeneities in the amino-acid replacement process. *Mol. Biol. Evol.* **21**, 1095–1109 (2004).

9. N. Lartillot, H. Brinkmann, H. Philippe, Suppression of long-branch attraction artefacts in the animal phylogeny using a site-heterogeneous model. *BMC Evol. Biol.* **7**, S4 (2007).

10. N. Lartillot, N. Rodrigue, D. Stubbs, J. Richer, Phylobayes mpi: Phylogenetic reconstruction with infinite mixtures of profiles in a parallel environment. *Syst. Biol.* **62**, 611–615 (2013).

11. A. M. A. Aguinaldo, J. M. Turbeville, L. S. Linford, M. C. Rivera, J. R. Garey, R. A. Raff, J. A. Lake, Evidence for a clade of nematodes, arthropods and other moulting animals. *Nature* **387**, 489–493 (1997).

12. J. Bergsten, A review of long-branch attraction. *Cladistics* **21**, 163–193 (2005).

13. J. E. Tarver, R. S. Taylor, M. N. Puttick, G. T. Lloyd, W. Pett, B. Fromm, B. E. Schirrmeister, D. Pisani, K. J. Peterson, P. C. J. Donoghue, Well-annotated microRNAomes do not evidence pervasive miRNA loss. *Genome Biol. Evol.* **10**, 1457–1470 (2018).

14. D. Papillon, Y. Perez, X. Caubit, Y. Le Parco, Identification of chaetognaths as protostomes is supported by the analysis of their mitochondrial genome. *Mol. Biol. Evol.* **21**, 2122–2129 (2004).

15. P. Natsidis, P. H. Schiffer, I. Salvador-Martínez, M. J. Telford, Computational discovery of hidden breaks in 28S ribosomal RNAs across eukaryotes and consequences for RNA integrity numbers. *Sci. Rep.* **9**, 19477 (2019).

16. O. Simakov, T. Kawashima, F. Marlétaz, J. Jenkins, R. Koyanagi, T. Mitros, K. Hisata, J. Bredeson, E. Shoguchi, F. Gyoja, J.-X. Yue, Y.-C. Chen, R. M. Freeman, A. Sasaki, T. Hikosaka-Katayama, A. Sato, M. Fujie, K. W. Baughman, J. Levine, P. Gonzalez, C. Cameron, J. H. Fritzenwanker, A. M. Pani, H. Goto, M. Kanda, N. Arakaki, S. Yamasaki, J. Qu, A. Cree, Y. Ding, H. H. Dinh, S. Dugan, M. Holder, S. N. Jhangiani, C. L. Kovar, S. L. Lee, L. R. Lewis, D. Morton, L. V. Nazareth, G. Okwuonu, J. Santibanez, R. Chen, S. Richards, D. M. Muzny, A. Gillis, L. Peshkin, M. Wu, T. Humphreys, Y.-H Su, N. H. Putnam, J. Schmutz, A. Fujiyama, J.-R. Yu, K. Tagawa, K. C. Worley, R. A. Gibbs, M. W. Kirschner, C. J. Lowe, N. Satoh, D. S. Rokhsar, J. Gerhart, Hemichordate genomes and deuterostome origins. *Nature* **527**, 459–465 (2015).

17. W. Wang, J. Zhong, B. Su, Y. Zhou, Y. Q. Wang, Comparison of Pax1/9 locus reveals 500-Myr-old syntenic block and evolutionary conserved noncoding regions. *Mol. Biol. Evol.* **24**, 784–791 (2007).

18. P. Kapli, M. J. Telford, Topology-dependent asymmetry in systematic errors affects phylogenetic placement of Ctenophora and Xenacoelomorpha. *Sci. Adv.* **6**, eabc5162 (2020).

19. M. J. Telford, G. E. Budd, H. Philippe, Phylogenomic insights into animal evolution. *Curr. Biol.* **25**, R876–R887 (2015).

20. K. Grobben, Die systematische einteilung des tierreichs. *Verhandlungen der Zool. Gesellschaft Österreich.* **58**, 491–511 (1908).

21. F. Marlétaz, E. Martin, Y. Perez, D. Papillon, X. Caubit, C. J. Lowe, B. Freeman, L. Fasano, C. Dossat, P. Wincker, J. Weissenbach, Y. Le Parco, Chaetognath phylogenomics: A protostome with deuterostome-like development. *Curr. Biol.* **16**, R577–R578 (2006).

22. P. Willmer, *Inverterbrate Relationships: Patterns in Animal Evolution* (Cambridge University Press, 1990).

23. J. M. Martín-Durán, R. Janssen, S. Wennberg, G. E. Budd, A. Hejnol, Deuterostomic development in the protostome *Priapulus caudatus*. *Curr. Biol.* **22**, 2161–2166 (2012).

24. S. A. Wennberg, R. Janssen, G. E. Budd, Early embryonic development of the priapulid worm *Priapulus caudatus*. *Evol. Dev.* **10**, 326–338 (2008).

25. E. E. Ruppert, Key characters uniting hemichordates and chordates: Homologies or homoplasies? *Can. J. Zool.* **83**, 8–23 (2005).

26. D. G. Shu, S. C. Morris, J. Han, L. Chen, X. L. Zhang, Z. F. Zhang, H. Q. Liu, Y. Li, J. N. Liu, Primitive deuterostomes from the Chengjiang Lagerstätte (Lower Cambrian, China). *Nature* **414**, 419–424 (2001).

27. Q. Ou, S. C. Morris, J. Han, Z. Zhang, J. Liu, A. Chen, X. Zhang, D. Shu, Evidence for gill slits and a pharynx in Cambrian vetulicolians: Implications for the early evolution of deuterostomes. *BMC Biol.* **10**, 81 (2012).

28. J. Han, S. C. Morris, Q. Ou, D. Shu, H. Huang, Meiofaunal deuterostomes from the basal Cambrian of Shaanxi (China). *Nature* **542**, 228–231 (2017).

29. S. C. Morris, S. L. Halgedahl, P. Selden, R. D. Jarrard, Rare primitive deuterostomes from the Cambrian (Series 3) of Utah. *J. Paleo.* **89**, 631–636 (2015).

30. K. J. Peterson, D. J. Eernisse, The phylogeny, evolutionary developmental biology, and paleobiology of the Deuterostomia: 25 years of new techniques, new discoveries, and new ideas. *Org. Divers. Evol.* **16**, 401–418 (2016).

31. A. Hejnol, M. Q. Martindale, Acoel development indicates the independent evolution of the bilaterian mouth and anus. *Nature* **456**, 382–386 (2008).

32. J. A. Cunningham, A. G. Liu, S. Bengtson, P. C. J. Donoghue, The origin of animals: Can molecular clocks and the fossil record be reconciled? *Bioessays* **39**, 1–12 (2017).

33. T. Flouri, A. Stamatakis, P. Kapli, newick-tools: A novel software for simulating and processing phylogenetic trees (2018); https://github.com/xflouris/newick-tools.

34. L. T. Nguyen, H. A. Schmidt, A. Von Haeseler, B. Q. Minh, IQ-TREE: A fast and effective stochastic algorithm for estimating maximum-likelihood phylogenies. *Mol. Biol. Evol.* **32**, 268–274 (2015).

35. K. Strimmer, A. Von Haeseler, Likelihood-mapping: A simple method to visualize phylogenetic content of a sequence alignment. *Proc. Natl. Acad. Sci. U.S.A.* **94**, 6815–6819 (1997).

36. N. Hamilton, M. Ferry, ggtern: Ternary diagrams using ggplot2. *J. Stat. Software, Code Snippets.* **87**, 1–17 (2018).

37. C. Camacho, G. Coulouris, V. Avagyan, N. Ma, J. Papadopoulos, K. Bealer, T. L. Madden, BLAST+: Architecture and applications. *BMC Bioinformatics.* **10**, 421 (2009).

38. L. S. Quang, O. Gascuel, N. Lartillot, Empirical profile mixture models for phylogenetic reconstruction. *Bioinformatics* **24**, 2317–2323 (2008).

39. D. A. Ludeman, N. Farrar, A. Riesgo, J. Paps, S. P. Leys, Evolutionary origins of sensation in metazoans: Functional evidence for a new sensory organ in sponges. *BMC Evol. Biol.* **14**, 3 (2014).

40. H. C. Wang, B. Q. Minh, E. Susko, A. J. Roger, Modeling site heterogeneity with posterior mean site frequency profiles accelerates accurate phylogenomic estimation. *Syst. Biol.* **67**, 216–235 (2018).

41. P. Simion, H. Philippe, D. Baurain, M. Jager, D. J. Richter, A. Di Franco, B. Roure, N. Satoh, É. Quéinnec, A. Ereskovsky, P. Lapébie, E. Corre, F. Delsuc, N. King, G. Wörheide, M. Manuel, A large and consistent phylogenomic dataset supports sponges as the sister group to all other animals. *Curr. Biol.* **27**, 958–967 (2017).

42. M. G. Grabherr, B. J. Haas, M. Yassour, J. Z. Levin, D. A. Thompson, I. Amit, X. Adiconis, L. Fan, R. Raychowdhury, Q. Zeng, Z. Chen, E. Mauceli, N. Hacohen, A. Gnirke, N. Rhind, F. di Palma, B. W. Birren, C. Nusbaum, K. Lindblad-Toh, N. Friedman, A. Regev, Trinity: Reconstructing a full-length transcriptome without a genome from RNA-Seq data. *Nat. Biotechnol.* **29**, 644–652 (2011).

43. Y. J. Luo, M. Kanda, R. Koyanagi, K. Hisata, T. Akiyama, H. Sakamoto, T. Sakamoto, N. Satoh, Nemertean and phoronid genomes reveal lophotrochozoan evolution and the origin of bilaterian heads. *Nat. Ecol. Evol.* **2**, 141–151 (2018).

44. D. Petit, E. Teppa, A. M. Mir, D. Vicogne, C. Thisse, B. Thisse, C. Filloux, A. Harduin-Lepers, Integrative view of α2,3-sialyltransferases (ST3Gal) molecular and functional evolution in deuterostomes: Significance of lineage-specific losses. *Mol. Biol. Evol.* **32**, 906–927 (2015).

45. T. F. M. Cummings, K. Gori, L. Sanchez-Pulido, G. Gavriilidis, D. Moi, A. R. Wilson, E. Murchison, C. Dessimoz, C. P. Ponting, M. A. Christophorou, Protein citrullination was introduced into animals by horizontal gene transfer from cyanobacteria. *bioRxiv* 10.1101/2020.06.13.150037 (2020).
